# A multimodal vision knowledge graph of cardiovascular disease

**DOI:** 10.1038/s44161-025-00757-4

**Published:** 2025-12-29

**Authors:** Khaled Rjoob, Kathryn A. McGurk, Sean L. Zheng, Lara Curran, Mahmoud Ibrahim, Lingyao Zeng, Vladislav Kim, Shamin Tahasildar, Soodeh Kalaie, Deva S. Senevirathne, Parisa Gifani, Vladimir Losev, Jin Zheng, Wenjia Bai, Antonio de Marvao, James S. Ware, Christian Bender, Declan P. O’Regan

**Affiliations:** 1 MRC Laboratory of Medical Sciences, Imperial College London, London, UK; 2 National Heart and Lung Institute, Imperial College London, London, UK; 3 Royal Brompton Hospital, London, UK; 4 Bayer AG, Research and Development, Pharmaceuticals, Wuppertal, Germany; 5 Department of Computing, Department of Brain Sciences and Data Science Institute, Imperial College London, London, UK; 6 British Heart Foundation Centre of Research Excellence, School of Cardiovascular and Metabolic Medicine and Sciences, King’s College London, London, UK; 7 Department of Women and Children’s Health, King’s College London, London, UK

**Keywords:** Cardiovascular diseases, Drug discovery

## Abstract

Understanding gene–disease associations is important for uncovering pathological mechanisms and identifying potential therapeutic targets. Knowledge graphs can represent and integrate data from multiple biomedical sources, but lack individual-level information on target organ structure and function. Here we develop CardioKG, a knowledge graph that integrates over 200,000 computer vision-derived cardiovascular phenotypes from biomedical images with data extracted from 18 biological databases to model over a million relationships. We used a variational graph auto-encoder to generate node embeddings from the knowledge graph to predict gene–disease associations, assess druggability and identify drug repurposing strategies. The model predicted genetic associations and therapeutic opportunities for leading causes of cardiovascular disease, which were associated with improved survival. Candidate therapies included methotrexate for heart failure and gliptins for atrial fibrillation, and the addition of imaging data enhanced pathway discovery. These capabilities support the use of biomedical imaging to enhance graph-structured models for identifying treatable disease mechanisms.

## Abstract (web summary)

Rjoob et al. develop CardioKG, a knowledge graph built on cardiac imaging traits to identify genetic associations and potential therapeutic strategies and drug repurposing opportunities for cardiovascular diseases.

## Main

Comprehension of gene–disease associations is crucial for deciphering the molecular mechanisms underlying various diseases and identifying potential therapeutic targets^1^. Knowledge graphs (KGs) have been used to systematically model and interrogate the biology regulating complex systems and diseases at multiple scales of organization^2^. A KG represents real-world facts and semantic relationships in a graph structure comprising nodes and edges. Entities within a KG represent genomics, transcriptomics, proteomics, molecular functions (MF), intra- and intercellular pathways, phenotypes, therapeutics and environmental exposures^3^. To construct a KG, information is aggregated from curated databases, nonstandardized repositories and evolving ontologies^4^. Machine learning approaches are applied to map entities and relationships to a low-dimensional vector space that represents its semantic structure more efficiently for downstream prediction tasks^5^.

While KGs provide a comprehensive framework for predicting potential gene–disease associations and prioritizing candidates for further investigation^4,6^, they lack individual-level phenotypes encoding target organ structure and function. In this work we introduce CardioKG, a KG that integrates rich computer vision-derived phenotypes of cardiovascular structure and function from biomedical imaging with data sourced from diverse biological databases. This approach leverages human ‘endophenotypes’, which are closer to the pathophysiology of disease than other observable traits, to improve the prediction of gene–disease associations. We achieve this through developing an embedding algorithm that preserves the intrinsic properties of nodes and their directional relationship. We assess the performance of this vision-augmented KG, using 21 different imaging traits along with data from 18 biomedical databases for the detection of gene–disease associations and drug repurposing with functional enrichment analysis to assess the prioritized genes’ role in critical biological pathways. This work shows how biomedical imaging possesses semantically meaningful information in multimodal graph-structured models of human disease for precision medicine applications.

## Results

### Study overview

The UK Biobank study recruited around 500,000 participants aged 40–69 years between 2006 and 2010^7^. A substudy recalled participants for cardiac magnetic resonance (CMR) imaging and computer vision analysis was subsequently used to measure 21 image-derived phenotypes capturing dynamic structural and functional systolic and diastolic traits of the ventricles, atria and aorta^8^. For building the KG we selected 4,280 participants who had imaging and a diagnosis of atrial fibrillation (AF), heart failure (HF), myocardial infarction (MI), hypertrophic cardiomyopathy (HCM) or dilated cardiomyopathy (DCM) (Supplementary Table 1), as well as a healthy group of 5,304 participants to capture a range of phenotypic diversity. The mean age at diagnosis was 67.6 ± 8.8 years and the mean age at CMR was 69.5 ± 6.6 years. The mean time from diagnosis to CMR was 1.95 ± 6.35 years. In total, over 200,000 image-derived phenotypes were used in the model. The results are presented for the three most prevalent and potentially treatable diseases in our cohort (AF, HF and MI). Baseline characteristics of the selected participants are summarized in Supplementary Table 2. A validation group of 489 participants without imaging was used for assessing drug repurposing outcomes.

**Fig. 1:**
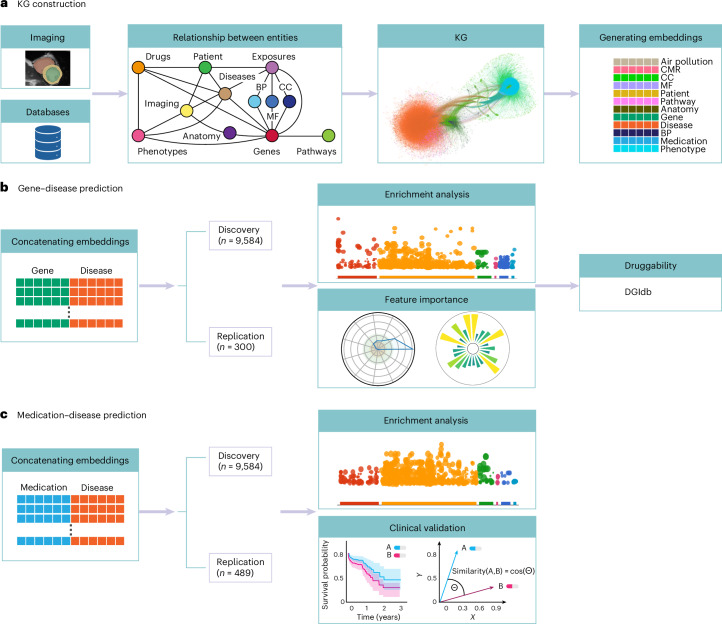
Overview of the KG construction and embedding process. Data were extracted from UK Biobank and 18 external databases to define entities (for example, genes, diseases, medications, pathways and imaging features) and their relationships. **a**, A schematic diagram illustrating the structure of the resulting multimodal KG. A DVGAE was then applied to learn low-dimensional embeddings for each node, preserving both topology and semantic relationships^4^. **b**, For predicting potential gene–disease associations, we used a ground truth of known pairs from the DisGeNET^12^ database. Concatenated embeddings were used to train a machine learning classifier. Pathway enrichment analysis and ranking of feature importance was performed and potential druggability assessed using the DGIdb^14^. **c**, Using the same approach, known medication–disease pairs from the DrugBank^13^ database were used to predict new therapeutic associations. Validation was performed using enrichment analysis, outcome analysis and graph-based methods. Source data

**Fig. 2:**
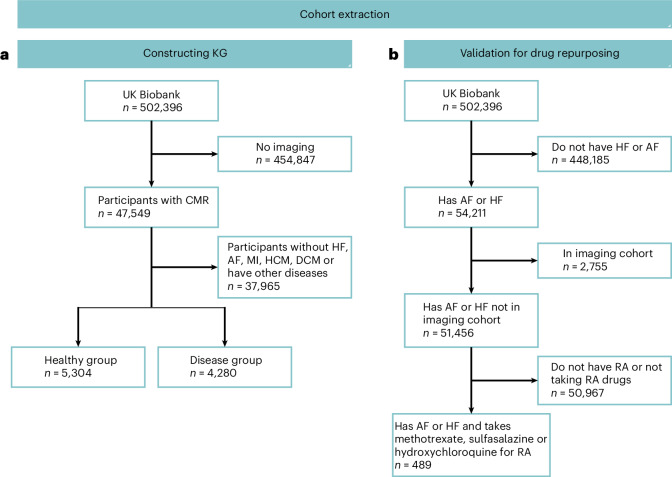
Cohort extraction from UK Biobank. **a**, The cohort (*n* = 9,548) used to construct the KG included participants with AF, MI, HF, HCM, DCM and healthy individuals. **b**, The group (*n* = 489) used to validate the predicted medications using survival analysis in drug repurposing, including those with AF or HF taking medication for RA.

**Fig. 3:**
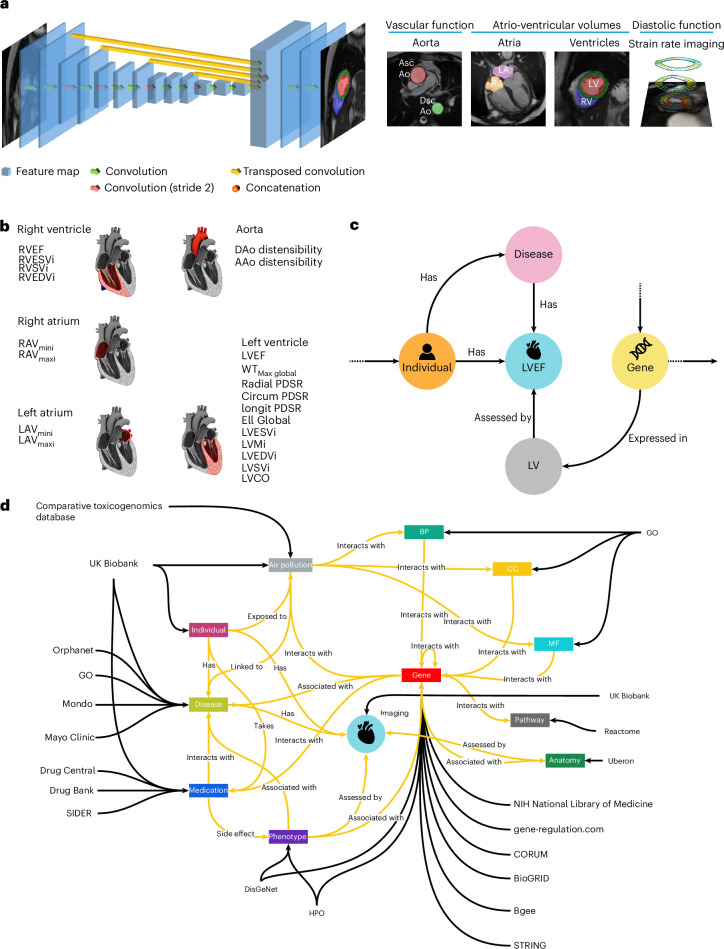
Integrating computer vision analysis to a KG. **a**, A fully convolutional network processes CMR images to output pixel-level segmentation maps. Motion analysis is also used to assess dynamic function of the heart and aorta. **b**, In total, 21 imaging traits were derived assessing structural and functional traits in the atria, ventricles and aorta. The corresponding anatomical regions for each trait were represented as nodes in the KG, allowing linkage of imaging-derived features with related diseases and phenotypes. **c**, Imaging features are integrated in the KG as nodes. A representative subgraph of the model is displayed, highlighting the integration of CMR-derived features within the KG. **d**, A schematic showing the relationships between the entities, the direction of the relationships and the source database for each entity. The imaging node is highlighted in the center of the KG. Ao, aorta; AAo, ascending aorta; DAo, descending aorta; Ell, global longitudinal strain; LAV, left atrium volume; LV, left ventricle; LVCO, left ventricular cavity obliteration; LVEDV, left ventricle ejection diastolic volume; LVESV, left ventricle ejection systolic volume; LVM, left ventricle mass; LVSV, left ventricle systolic volume; PDSR, peak diastolic strain rate; RAV, right atrium volume; RV, right ventricle; RVEDV, right ventricle ejection diastolic volume; RVEF, right ventricle ejection fraction; RVESV, right ventricle ejection systolic volume; RVSV, right ventricle systolic volume; Asc, ascending; Dsc, descending; i, indexed; WT, wall thickness. Panel **a** reproduced from ref. ^8^ under a Creative Commons license CC-BY 4.0.

We constructed CardioKG, a KG that integrates cardiovascular image-derived traits with external biomedical databases to model the complex biological relationships between genes, diseases and phenotypes. We then trained a variational graph auto-encoder (VGAE)^9^ to generate node embeddings from the resulting KG using an approach we developed to preserve directional relationships, enabling the application of machine learning models to more accurately predict gene–disease associations. Functional enrichment^6^ analysis was used to identify related molecular mechanisms, biological processes and pathways. A druggability analysis^10^ of the predicted genes evaluated therapeutic potential. Finally, we conducted a drug repurposing^11^ analysis to identify existing medications that could be repositioned to target the diseases of interest. Figure 1 shows the overall design of the study and Fig. 2 shows how the participants were selected. Figure 3 shows how computer vision-derived phenotypes were connected to other entities in the KG.

Ground truth labels for training a machine learning classifier to predict potential gene–disease and medication–disease pairings were obtained from DisGeNET^12^ and DrugBank^13^ databases. Associations not used in the construction of CardioKG were used for training. The embeddings were concatenated to preserve information from each model. Enrichment analysis was conducted for the top ten predicted genes, and we assessed the importance of related entities and imaging features. Druggability was evaluated using the Drug–Gene Interaction database (DGIdb)^14^.

### Data extraction and KG construction

In addition to the imaging data, we aggregated information from 18 biomedical databases (Methods) to extract relevant knowledge on gene names, disease names, medication names, interactions between the medications and genes and other related biomedical terms, and converted the extracted data into a graph structure of nodes and relationships (Fig. 3c). A total of 33,277 nodes and 1,195,437 relationships were extracted to construct CardioKG. The nodes included genes (*n* = 18,606), human participants (*n* = 9,584), medications (*n* = 2,106) and molecular pathways (*n* = 1,707). Phenotypic abnormalities were linked to human diseases (*n* = 1,036) and anatomical regions (*n* = 160), which included 5 structures segmented on imaging measuring 21 traits across each selected disease. Databases also included information on air pollutants, biological processes (BP), MF and cellular components (CC).

### Embedding algorithm

KG embeddings offer a compact numerical representation of the graph’s structure and content, enabling algorithms to learn and reason over the data. Each node (entity) and edge (relationship) is assigned a vector representation through minimizing a loss function. Here, we developed an architecture based on a directed variational graph auto-encoder (DVGAE), which generates embeddings of the KG while preserving node and relationship properties as well as the directionality of relationships. The reconstructed graph accurately represented the original graph with high edge-wise accuracy of the embeddings (98.1%). Alternative methods such as Node2Vec^15^, TransE^16^ and ComplEx^17^ do not capture node and edge-specific features or directionality, which are attributes of our KG structure.

### Predicting gene–disease associations

Embeddings of genes associated or not associated with disease were concatenated with the embeddings of each disease of interest (Supplementary Tables 3–5). Ground truth labels for training used DisGeNET, which is a database containing over 2 million gene–disease associations, involving 29,000 genes and over 42,000 diseases with 4.3 million variant–disease associations^12^ aggregated from a dozen repositories that annotate clinically relevant variants (ClinVar) or genes (ClinGen and Genomics England PanelApp, among others). Three machine learning algorithms—random forest, support vector machine (SVM) and artificial neural networks (ANNs)—were trained on the concatenated embeddings to predict the association between the genes and disease using fivefold cross-validation. SVM achieved the best performance based on accuracy (72.4% in HF, 75.0% in AF and 83.3% in MI), specificity (93.0% in HF, 97.0% in AF and 91.7% in MI), sensitivity (52.0% in HF, 51.6% in AF and 69.2% in MI) and area under the receiver operating characteristic curve (AUC–ROC, 0.80 in HF, 0.78 in AF and 0.83 in MI) (Supplementary Table 6). Hence, SVM was used to predict potential associations for unlabeled genes without a known disease gene–disease association. Each predicted gene was assigned and ranked by a predicted probability of its association with disease. The top ten predicted genes (Supplementary Table 7) were selected as examples for functional enrichment analysis. A comparison was made for pathway enrichment using a KG without imaging traits. A hypergeometric test was used to determine whether a set of predicted genes is statistically overrepresented in a predefined reference gene set.

The top ten predicted genes for HF included *GATA2*, *AGR1* and *EP300*, which were significantly associated with 815 pathways (Supplementary Fig. 1 and Supplementary Information), among which 66 were identified as relevant pathways such as angiogenesis and the MAPK cascade (Supplementary Fig. 2). These findings implicate genes in signaling cascades that regulate cellular regeneration and aging as potential genetic modifiers of HF. Variants in the MAPK pathway have also recently been implicated in genome-wide association study (GWAS) data not used for KG training^18^. Without imaging traits, only four relevant pathways were identified (Supplementary Fig. 2 and Supplementary Table 7).

In AF, enrichment for prioritized genes including *SRC*, *GATA1* and *HSPA8* was identified in 658 pathways, of which 14 were relevant pathways associated with AF, including processes regulating cardiac conduction, response to hypoxia and regulation of immune system process (Supplementary Fig. 3 and Supplementary Information). These findings implicate genes that may regulate immune response and inflammation in AF, emerging as risk factors for arrhythmic disease^19^, and support a potential role for *SRC*, which has been proposed as a promising target in other cardiovascular diseases^20^. Without imaging traits, only one relevant pathway for AF was identified (Supplementary Table 7 and Supplementary Fig. 3).

Last, in MI, predicted genes included *PCNA*, *HTT* and *SNCA*, which were significantly associated with 406 pathways, including 42 relevant pathways related to MI, such as apoptosis and cellular response to stress (Supplementary Fig. 4 and Supplementary Information). Enrichment analysis without CMR features revealed only four relevant pathways associated with MI (Supplementary Table 7 and Supplementary Fig. 4).

### Druggablity of predicted genes

We assessed the therapeutic potential of the top ten predicted genes for each disease using the DGIdb, which curates information on drugs known to inhibit, activate or otherwise modulate the activity of specific genes or their protein products. This evaluation focused on determining whether genes prioritized by CardioKG are actionable by existing drugs. Among the top ten predicted genes, defined as genes not previously reported as significantly associated with HF, AF or MI according to DisGeNET, a comprehensive gene–disease association database integrating information from ClinVar, GWAS Catalog, UniProt, Orphanet and published literature, five were identified as druggable (*AR*, *APP*, *GATA2*, *EGR1* and *EP300*). These genes have been recognized as potential therapeutic targets and can be modulated by a total of 48 medications. Drugs including the monoclonal antibodies ponezumab and bapinezumab have been identified as potential candidates for targeting *APP* for instance (Supplementary Fig. 5).

For predicted genes associated with AF, seven were druggable (*SRC*, *CASP8*, *DAPK1*, *H2AX*, *HSPA8*, *EP300* and *HNF4A*). These genes can be targeted by 37 medications including several antidiabetic ‘gliptins’ (dipeptidyl peptidase-4 inhibitors), which have observational evidence of a potential anti-arrhythmic role in treated patients with diabetes (Supplementary Fig. 6)^21^. Finally for MI, of the predicted genes, two were identified as druggable (*SNCA* and *H2AX*). These genes can be targeted by four medications, including apoptosis inducers eltanexor and selinexor (Supplementary Fig. 7). Without CMR features in the KG, only two genes were identified as potentially druggable for each of HF (Supplementary Fig. 8), AF (Supplementary Fig. 9) and MI (Supplementary Fig. 7).

### Importance of image-derived phenotypes

**Fig. 4:**
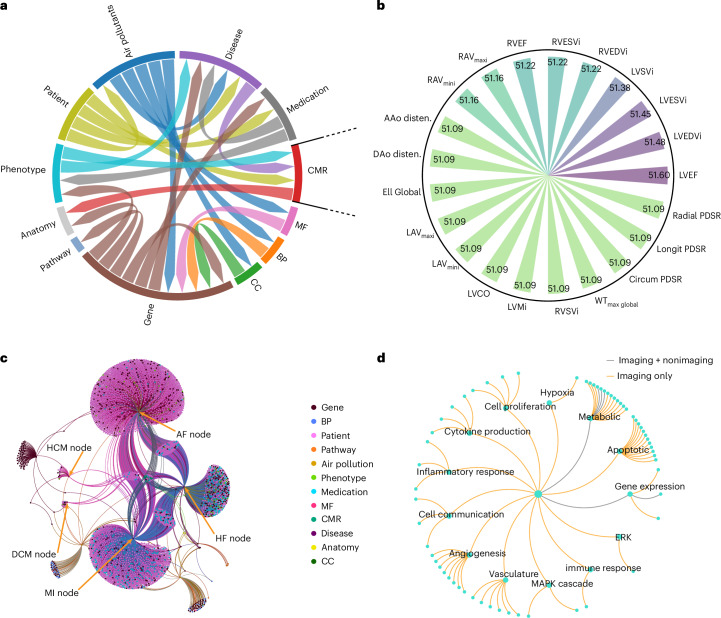
Contribution of cardiovascular imaging features to the structure and inference of the KG. An illustration of how CMR features integrate into the KG and influence its structure and downstream analyses. The aim is to assess the relative importance of CMR-derived imaging parameters to evaluate whether including imaging improves pathway discovery. **a**, A circos plot shows the extracted entities and the directional relationships between them, serving as a compact visual summary of the underlying KG structure. **b**, PageRank scores for each imaging parameter (*n* = 21) within the KG indicating their relative influence and connectivity to other influential nodes. LVEF ranked highest, followed by right ventricular and atrial parameters. Other values, including aortic function and strain shared similar scores. **c**, Network interactions among genes, diseases, pathway, medications and CMR features. **d**, Enriched pathways identified with and without the use of imaging. Pathways highlighted in orange are specific to analyses using imaging data only, whereas those in gray represent pathways that were enriched whether imaging data were used or not. ERK, extracellular signal-regulated kinase; disten, distensibility.

The importance of encoded imaging traits was assessed by PageRank^22^, which quantifies node centrality based on the structure of incoming connections, assigning higher scores to nodes connected to other highly ranked nodes (Supplementary Fig. 10). The CMR entity had the highest score (51.09–51.60) reflecting imaging’s central position within the graph and connection density to other node types (Fig. 4) with left ventricular ejection fraction (LVEF) the highest ranked feature.

To evaluate their structural role, we conducted an ablation experiment in which CMR nodes were removed from the graph. This resulted in decreased performance of the SVM model (Supplementary Table 6). The CMR nodes function as intermediaries linking anatomical features to genetic and disease-level data with their removal affecting graph connectivity, which impairs the model’s capacity to capture indirect associations. This leads to predictions that are less aligned with disease-relevant pathways. Removing environmental variables such as air pollution did not alter the composition of the top ten predicted genes for HF. The same genes were identified before and after exclusion of environmental nodes, with only minor differences in ranking order (Supplementary Table 8). This consistency indicates that the model’s predictions are robust, and that environmental factors, while highly connected in the graph, do not solely drive the gene–disease associations.

The number of relevant pathways identified for diseases of interest is also greater when CMR features are included (*χ*^2^, *P* = 0.001). Together these findings indicate that the inclusion of imaging nodes enhances the KG’s structural and functional complexity, enabling the model to better leverage interconnected biological data to uncover genes linked to a broader range of critical pathways.

### Minimal model configuration

The minimal model configuration retained strong predictive performance. This excluded five entities with lower PageRank scores (pathway 0.6; BP, 0.7; CC, 0.7; MF, 0.7; phenotype, 0.9), but the top ten predicted genes for HF remained the same as in the full model, with only minor differences in ranking (Supplementary Fig. 11). Furthermore, the performance based on AUC–ROC was 0.80 for the full entity set and 0.78 for the minimal configuration. This indicates that the predictive power of the model is preserved by the core entity set (disease, gene, exposure, medication, anatomy and CMR).

### Drug repurposing

Embeddings of the diseases of interest and medications either indicated or contraindicated for them were concatenated and used to train a model to predict potential disease–medication associations. The predicted medications were further evaluated through enrichment analyses of their target genes, survival analysis and graph-based validation.

**Fig. 5:**
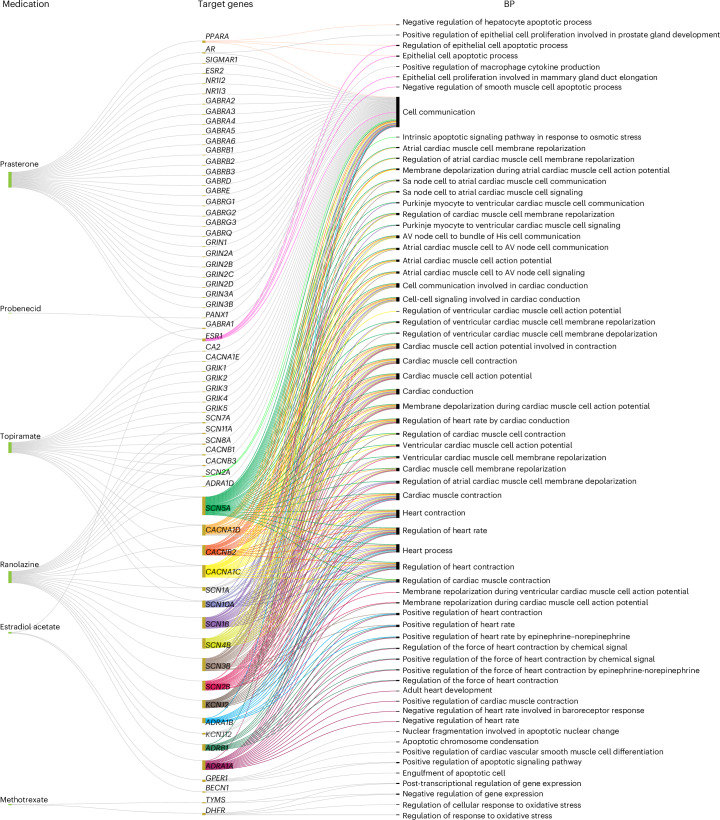
Enrichment analysis of target genes for predicted medications treating HF. An enrichment analysis was conducted to identify critical pathways for HF that are significantly associated with the target genes. Illustrated are the predicted medications for HF, their associated target genes and the enriched pathways linked to those genes. This figure excludes associated nonpharmaceutical substances (glutamic acid, caffeine and cannabidiol). The same enrichment analysis was carried out for AF and MI (Supplementary Figs. 15 and 16). Sa, sinoatrial; AV, atrioventricular.

The KG-based machine learning model identified potential associations between HF and a range of existing medications. Among these, the top ten candidates included methotrexate, topiramate and ranolazine, prioritized based on their predicted association scores (Supplementary Table 9). Pathway enrichment analysis of the predicted medications was performed using known target genes. The target of methotrexate, *DHFR*, was linked to regulation of oxidative stress response, which is a key factor in the pathophysiology of HF and cardiac remodeling^23^. In addition, the targets of topiramate, including *SCN5A*, *SCN10A*, *CACNA1C* and *CACNA1D*, showed significant associations with essential HF pathways, such as myocyte contraction and action potential regulation. The full enrichment analysis results are shown in Fig. 5.

A subset of UK Biobank participants, not included in model development, was used to determine whether any of the predicted medications were associated with improved survival outcomes in HF. Methotrexate was the only candidate included in the survival analysis owing to the limited number of individuals exposed to most of the predicted medications. Here participants diagnosed with HF and rheumatoid arthritis (RA) were considered (*n* = 181), since RA is a common indication for the use of methotrexate. Three subgroups were identified based on treatment with commonly prescribed RA medications: (1) patients with RA taking methotrexate (*n* = 121), (2) patients with RA taking sulfasalazine (*n* = 27) and (3) patients with RA taking hydroxychloroquine (*n* = 33) (Supplementary Table 10). All three medications are disease-modifying antirheumatic drugs routinely used for the management of RA. Patients with HF also being treated with methotrexate for RA had higher survival probability (hazard ratio 0.65 (95% confidence interval 0.48–0.89), *P* < 0.01) compared to those treated with hydroxychloroquine and sulfasalazine (Supplementary Fig. 12 and Supplementary Table 11).

To externally validate these findings, we analyzed data from the BioVU biorepository holding information on over 300,000 participants^24^. We assessed patients with both HF and RA (*n* = 6,876). To ensure unbiased analysis we performed propensity matching using the R-package MatchIt^25^. After this, we used participants being treated with methotrexate (*n* = 274) or hydroxychloroquine (*n* = 200) and sulfasalazine (*n* = 16). As an endpoint we defined an ‘adverse event’ as all-cause death or reported class 3 or higher in the New York Heart Association classification system. Methotrexate showed a significantly higher survival probability (hazard ratio 0.71 (95% confidence interval 0.5–0.99), *P* < 0.05), supporting the observation from UK Biobank (Supplementary Table 12 and Supplementary Fig. 13). A recent large-scale registry analysis of 900,000 adult patients with RA in the TriNetX Diamond network reported that methotrexate use was associated with fewer cardiovascular events, especially congestive HF, compared to untreated propensity-matched RA patients^26^.

The medications currently approved for HF and the KG-predicted medications had high cosine similarity scores (Supplementary Fig. 14), which indicates similar vector representations, suggesting they share similar relational or structural contexts within the graph reinforcing their potential therapeutic relevance (Supplementary Fig. 14).

A similar analysis was conducted for AF, where the top ten candidate medications, including methotrexate, zonisamide, acamprosate and probenecid, were prioritized (Supplementary Table 13). Enrichment analysis revealed that the target of methotrexate, *DHFR*, is also involved in cellular pathways that promote AF^27,28^. In addition, the targets of zonisamide, including *SCN5A*, *SCN4B* and *SCN1B*, are associated with relevant pathways in AF, such as the regulation of cardiac muscle cell membrane repolarization and depolarization (Supplementary Fig. 15). A similar survival analysis was performed in patients with both RA and AF being treated with methotrexate and alternative drugs (Supplementary Table 14), but no significant differences in survival probability were observed (Supplementary Fig. 17 and Supplementary Table 15). Network-based validation revealed that the predicted medications shared high cosine similarity with those already indicated for AF, supporting their therapeutic relevance and biological plausibility (Supplementary Fig. 18).

For MI the ten highest-ranking drug candidates, selected based on their prediction scores, were prioritized (Supplementary Table 16) for pathway enrichment analysis in the same way. This included vorinostat, which targets HDAC6 in the regulation of cellular responses to oxidative stress and the regulation of reactive oxygen species metabolism (Supplementary Figs. 16 and 19). Survival analysis was not performed as these drugs are not commonly prescribed. A graph-based validation also indicated high cosine similarity between the predicted medications and those already approved for MI (Supplementary Fig. 20).

### Independent validation of predictions

We evaluated the reproducibility of our findings in an independent UK Biobank cohort of 33,822 participants with CMR imaging, including 1,173 with AF, 756 with MI, 274 with HF, 25 with HCM and 34 with DCM (Supplementary Table 17). In this cohort, methotrexate was the highest ranked predicted medication for HF (Supplementary Table 18). Similarly, the same top ten genes were predicted to be associated with HF with only changes in the ranking of these (Supplementary Table 19). We also excluded patients with comorbidities (hypertension n = 20, diabetes n = 7, chronic kidney disease (*n* = 11) and chronic obstructive pulmonary disease (*n* = 15)), yielding the same top predicted genes, with only minor changes in ranking (Supplementary Table 20).

## Discussion

A KG is a semantic network representing the relationship between diverse real-world biomedical entities to enable systematic study of human disease. CardioKG provides a holistic view of cardiovascular disease by integrating data across 19 diverse biological databases representing relationships between genomics, molecular pathways and exposures. Here, we show how precision phenotypes, abstracted from computer vision analysis of the heart and circulation, leverage such rich interconnected biological data to uncover potential candidate genes and therapeutics linked to three major causes of morbidity and mortality worldwide^29^. We also demonstrate the potential of predicted drug repurposing to improve patient outcomes in HF. Together this shows how the performance of disease-specific semantic models can be substantially improved using cardiovascular imaging.

GWASs find associations between common variants and disease^30^, but have limitations in identifying causal mechanisms, rely on single traits and do not leverage prior knowledge. While recent efforts using machine learning, such artificial intelligence-based summarization, aim to improve GWAS interpretation^31^, most associated variants still have individually small effects on traits or diseases and rarely indicate actionable targets. In contrast, KGs have emerged as a powerful framework for integrating data across multiple domains to predict potential gene–disease associations^32–34^. An important bottleneck has been the limited availability of individual-level phenotypes that can be linked to other semantic information in the network. Through leveraging advances in image segmentation and motion tracking in large biobank populations with MRI, we show how a KG network can be enriched by quantitative organ phenotypes. In CardioKG, a node for each image-derived phenotype is semantically linked to nodes encoding anatomical sites, genes and diseases. We show how this offers greater pathway enrichment for discovered genes, a higher yield of potentially druggable targets and biologically plausible predictions for drug repurposing. We also introduce a framework that generates KG embeddings while preserving directionality in node and relationship properties achieving high reconstruction fidelity.

Taking HF as an example, CardioKG prioritized genes linked to regulatory pathways that include apoptosis, angiogeneisis, inflammation and tissue hypoxia. One prioritized gene is *APP*, which is associated with amyloidogenic pathways in Alzheimer’s disease, but this multi-organ condition is also associated with systemic inflammation and oxidative stress affecting peripheral organs including the heart, probably associated with *Aβ* amyloid deposition^35^. The KG predicted several pharmaceutical compounds interacting with *APP* as agonists, inhibitors or modulators. Among these, the KG also predicted humanized monoclonal antibodies as potential therapeutics targeting *APP*^36,37^. The KG proposed methotrexate, topiramate and ranolazine for drug repurposing in HF with enriched associations for myocardial contraction and action potential regulation. Methotrexate also showed a potential survival benefit in patients with both HF and RA compared to other treatments. A trial of methotrexate to evaluate its cardiovascular benefits in patients with RA is underway^38^ and it has a favorable repurposing safety profile and cost-effectiveness^39^. Its benefits are independent of reductions in RA disease activity suggesting the KG has identified alternative methotrexate-related mechanisms in modulating cardiovascular risk^40^. The KG also prioritized anti-epiletic therapies that target *SCN5A*-regulated ion channels in the conduction system as treatments for AF, and vorinostat as a repurposed treatment for MI that targets *HDAC6* regulation of mitochondrial biogenesis^41^.

The limitations of this work are that the UK Biobank population is primarily of European descent highlighting the need for enriching the KG image phenotypes with more diverse populations. Participants in the imaging substudy of UK Biobank were invited at random^42^. The population is subject to healthy volunteer selection bias but still enables valid scientific inferences of associations between exposures, genetic variation and health conditions that are generalizable^43^. We considered all causes of HF for model training as coded subtypes had low prevalence in our population. We could not therefore predict associations specific to HF with preserved ejection fraction. This condition is also recognized as an aggregation of several distinct pathobiological entities^44^. Technologies for acquiring knowledge from multiple sources and integrating them into a KG structure are still developing, and there are technical challenges for graph completion, knowledge fusion and efficient reasoning that are areas of active development^45^. There are no established standards yet for graph-based biomedical data models but these may emerge as their use becomes more widespread. The use of large language models may complement the ability to acquire knowledge from unstructured text but, on their own, may give inaccurate or inconsistent results in biomedical knowledge discovery^46^.

Our findings point to the potential for vision-based KGs, that capture phenotypes closely coupled to underlying individual-level pathophysiology, to accelerate the discovery of potential therapeutics in cardiovascular science as well as medicine more broadly. Biomedical imaging encodes additive information in semantic networks and the versatile architecture of CardioKG is generalizable across multiple disease use-cases where imaging is available. Future opportunities may lie in personalizing diagnostic strategies through leveraging larger and more diverse population datasets.

## Methods

The UK Biobank cohort study recruited over 500,000 participants aged 40–69 years old from across the UK between 2006 and 2010^7^. The study received ethical approval from the National Research Ethics Service (11/NW/0382) and all participants gave written informed consent. This research has been conducted under application 40616. To construct the KG, we included 4,280 individuals who had both imaging data and a documented diagnosis of AF, HF, MI, HCM or DCM, based on ICD-9 and ICD-10 codes (Supplementary Table 1). In addition, a reference group of 5,304 healthy participants was incorporated to ensure a broad representation of phenotypic variability. However, our primary emphasis was placed on the three most common conditions: HF, AF and MI.

### Image-derived cardiovascular phenotypes

CMR imaging was performed on participants to capture two-dimensional retrospectively gated cine imaging on a 1.5T magnet (Siemens Healthineers)^47^. Cine images were acquired in the left ventricular short axis from base to apex, as well as in the long-axis two- and four-chamber views. Each cine series consisted of 50 cardiac frames, with a typical temporal resolution of 31 ms. Additional transverse cine images of the ascending and descending thoracic aorta were also obtained.

The two-dimensional cine images in both short- and long-axis views were segmented using fully convolutional neural networks, achieving segmentation quality comparable to that of expert human readers^8^ (Fig. 3). From these segmentations, quantitative measurements of the left and right ventricles were obtained, including end-diastolic volume, end-systolic volume, stroke volume and ejection fraction. Left ventricular mass was computed by multiplying myocardial volume by a density of 1.05 g ml^−1^.

Atrial volumes were calculated using the biplane area-length method, where *A*_2Ch_ and *A*_4Ch_ represent the atrial areas measured in the two- and four-chamber cine views, respectively, and *L* denotes the average longitudinal diameter across both views. The formula used was $V=\frac{8}{3\pi }\times \frac{{A}_{{\mathrm{2Ch}}}\times {A}_{{\mathrm{4Ch}}}}{L}.$

All CMR-derived measurements were indexed to body surface area (BSA), which was calculated using the Du Bois formula BSA = 0.007184 × height^0.725^ × weight^0.425^, with height in cm and weight in kg. Left ventricular wall thickness was measured at end diastole as the distance between the segmented epicardial and endocardial borders.

Circumferential and radial strains were computed from cine short-axis images using the formula ${E}_\mathrm{dir}=\frac{\Delta {L}_{{\mathrm{dir}}}}{{L}_{{\mathrm{dir}}}}$, where dir represents either the circumferential or radial direction, *L*_dir_ is the initial length of a line segment in that direction and *ΔL*_dir_ denotes the change in length over time. Longitudinal strain was estimated from motion tracking in the long-axis four-chamber view. Segmentation of the aortic cine images was performed using a spatiotemporal neural network^48^. From these segmentations, the maximum and minimum cross-sectional areas were extracted. Aortic distensibility was then calculated using central blood pressure values, obtained via peripheral pulse wave analysis (Vicorder)^49^.

### Data extraction

Data from participants in UK Biobank included CMR, disease records, air pollution exposure, medication history, age, gender, ethnicity and body mass index (BMI). These variables represent a comprehensive dataset capturing both phenotypic and environmental factors, allowing for a holistic analysis of the relationships between genetic predispositions, lifestyle factors and disease outcomes. CMR imaging provides detailed structural and functional cardiovascular parameters, which assess cardiac volumes and mass, diastolic function and aortic distensibility. Inclusion criteria for the KG experiments and outcome analysis are shown in Fig. 2. Eighteen external databases were used in the KG spanning a variety of biomedical domains including genetic annotations, protein interactions, pathway enrichment, disease ontologies and drug–target interactions. Details of the external databases are given below.

#### GO

Gene Ontology (GO) is a comprehensive and widely used resource that provides a standardized framework for representing the attributes of genes and gene products across all species^50^. Developed to unify biological terminology, it serves as a tool for organizing and interpreting vast amounts of genomic and proteomics data. GO categorizes biological knowledge into three main domains: MF, which describes the specific activities of a gene product, such as enzyme activity or binding affinity; BP, which represents the broader series of events or pathways that the gene or gene product contributes to, such as cell division or signal transduction; and CC, which indicates the specific locations within a cell where a gene product is active, such as the nucleus, membrane or extracellular region.

#### HPO

The Human Phenotype Ontology (HPO) provides a structured and standardized vocabulary to describe human phenotypic abnormalities, linking them to underlying genetic causes^51^. It is a resource for integrating phenotypic information across different datasets, facilitating the systematic analysis of phenotype–genotype correlations. The HPO framework is organized hierarchically, capturing phenotypic abnormalities at varying levels of granularity, from broad categories, such as ‘abnormality of the cardiovascular system’ to specific terms, such as ‘atrial septal defect’. Its curated dataset integrates knowledge from diverse sources, including clinical reports, literature and existing biomedical ontologies.

#### Bgee

Bgee is a comprehensive database of gene expression data across multiple species, offering a curated and standardized resource for comparative transcriptomics and functional genomics^52^. Bgee integrates gene expression data derived from multiple experimental platforms, including RNA-sequencing, microarrays and in situ hybridization, ensuring representation of gene expression patterns across diverse biological contexts. For this study, we retrieved gene expression data for humans from Bgee to provide insights into tissue- and organ-specific gene expression profiles. Bgee employs ontologies such as the Uberon multispecies anatomy ontology and developmental stage ontologies to annotate gene expression data systematically. This facilitates cross-species comparisons to investigate the evolutionary conservation of gene expression patterns in homologous tissues and organs. The integration of these ontologies also supports the exploration of gene functions within their anatomical and developmental contexts, providing deeper insights into the roles of genes in complex biological systems.

#### CTD

The Comparative Toxicogenomics Database (CTD) is a comprehensive resource that connects information about the interactions between chemicals, genes and diseases, enabling studies on chemical-induced diseases and toxicogenomic relationships through curated and integrated data^53^. By providing manually curated information from scientific literature, the database facilitates the exploration of complex molecular mechanisms underlying the effects of environmental exposures on health. The database also supports the identification of potential therapeutic targets and biomarkers by linking chemical exposures to specific genes, pathways and diseases. The database organizes information into structured relationships, such as chemical–gene interactions, chemical–disease associations and gene–disease associations. Furthermore, CTD includes information about chemical properties, dose–response relationships and exposure contexts.

#### DisGeNET

DisGeNET is a comprehensive knowledge platform that integrates data on human gene–disease associations from diverse sources, including expert curation, data from the scientific literature, databases of genetic variants and computational predictions, to support research in genomics and disease biology (version 24.4)^12^. As one of the most extensive repositories for gene–disease relationships, DisGeNET provides a unified framework to explore the genetic underpinnings of human diseases, encompassing both Mendelian and complex diseases, as well as rare conditions. The platform organizes its data into a structured and standardized format, enabling integration with other resources and tools. DisGeNET’s content is derived from a wide array of sources, such as ClinVar, GWAS Catalog, UniProt and Orphanet, and includes both experimentally validated associations and computationally inferred links. This diversity ensures a comprehensive dataset, offering insights into the genetic basis of diseases from multiple perspectives.

#### DrugBank

DrugBank (version 5.1.8) is a comprehensive and richly curated resource that integrates detailed drug data with extensive information on their mechanisms of action, interactions, targets and associated pathways^13^. Serving as a tool for drug development, pharmacological research and clinical applications, DrugBank integrates chemical, pharmacological and molecular biological data. The database bridges chemistry and biology by linking drugs to relevant proteins, genes and pathways, enabling researchers to study the complex interplay between therapeutics and biological systems. DrugBank includes information on a wide range of drug types, including small molecules, biologics, nutraceuticals and experimental compounds. For each drug, the database provides comprehensive annotations, including chemical structures, pharmacokinetics, mechanisms of action, drug–drug interactions and adverse effects. In addition, it contains detailed data on drug targets, enzymes, transporters and carriers, along with their sequences, structures and roles in human physiology. This supports applications such as drug repurposing, biomarker discovery and precision medicine.

#### DrugCentral

DrugCentral is a comprehensive resource that provides up-to-date, detailed information on FDA-approved drugs, including their indications, mechanisms of action, pharmacological properties and therapeutic uses^54^. It is designed to support a wide range of pharmaceutical research and development activities, including drug discovery, repurposing and repositioning studies. DrugCentral facilitates the identification of promising drug candidates, the exploration of potential therapeutic indications and the optimization of existing treatments. DrugCentral includes extensive information on the chemical, pharmacokinetic and pharmacodynamic profiles of FDA-approved drugs. For each drug, the database provides details such as chemical structure, molecular weight, drug classification, routes of administration and therapeutic indications. In addition, the resource offers insights into the mechanisms of action, targets and molecular pathways involved, enabling a deeper understanding of how drugs exert their effects at the molecular and cellular levels.

#### Mayo Clinic

Information linking symptoms, causes, risk factors, complications and prevention of 2,227 diseases and conditions was reused from publicly available web-scraped data from the Mayo Clinic knowledge base^4^.

#### MONDO disease ontology

MONDO is a unified disease ontology that integrates a wide array of disease classification systems, offering a harmonized framework for disease annotations and biomedical research^55^. By incorporating and standardizing information from diverse resources, MONDO facilitates consistent and comprehensive disease categorization, improving the interoperability and integration of data across various research fields. This integrative approach enhances the ability to link disease phenotypes to underlying genotypes, enabling a deep understanding of disease mechanisms and their genetic foundations. MONDO provides a standardized terminology for disease classification that encompasses a wide range of diseases, from rare genetic disorders to common multifactorial conditions.

#### Orphanet

Orphanet is a comprehensive reference portal dedicated to rare diseases and orphan drugs^56^. It provides an extensive collection of resources that include disease descriptions, diagnostic criteria, genetic underpinnings, epidemiology and clinical management guidelines. The portal facilitates the identification and understanding of rare conditions, which are often overlooked owing to their low prevalence. The database also includes detailed information on orphan drugs, which are critical for treating rare diseases, and offers access to regulatory information, market authorization and clinical usage.

#### Reactome

Reactome is an open-source pathway database that provides detailed information about the molecular mechanisms underlying biological processes^57^. It offers comprehensive insights into cellular pathways, their components and how these pathways interconnect with various physiological and pathological states. By mapping the flow of biological information across different molecular events, Reactome enables a deep understanding of cellular processes, such as signal transduction, gene expression, metabolism and immune response, as well as their associations with diseases. Reactome contains curated pathway annotations, which are continuously updated to reflect current research and experimental data. The database includes a wide variety of biological pathways, spanning simple molecular interactions to complex multistep processes involving various biomolecules, such as proteins, lipids and nucleic acids. These pathways are not only specific to humans but also cover pathways from model organisms, allowing for cross-species comparisons and a broader understanding of conserved biological mechanisms.

#### SIDER

The Side Effect Resource (SIDER, version: 4.1) is a comprehensive database that provides structured information on the side effects of marketed drugs, offering insights into drug safety and pharmacovigilance^58^. The data are compiled from publicly available information, including package inserts, clinical trial reports and postmarketing surveillance data. SIDER contains detailed records of side effects associated with a wide range of FDA-approved drugs, capturing both common and rare adverse events. The database includes information on the frequency, severity and type of side effects, as well as the specific patient populations that may be more vulnerable to these adverse reactions.

#### Uberon

Uberon is an integrated, cross-species ontology that provides a comprehensive and standardized description of anatomical structures across a wide range of organisms^59^. Uberon facilitates the integration of anatomical knowledge from different species, enabling researchers to explore the similarities and differences in the structures of various organisms. This ontology bridges the gap between species, allowing for a unified understanding of biological form and function. Uberon is designed to be a multispecies resource, covering not only human anatomy but also the anatomy of model organisms (such as mice, zebrafish and fruit flies) and nonmodel species, including plants and microorganisms. This broad scope allows for the comparison of anatomical structures across species, providing insights into conserved features, evolutionary adaptations and the functional roles of specific organs and tissues.

#### STRING

STRING (version:11.5) is a comprehensive database that provides detailed information on known and predicted protein–protein interactions, integrating an array of data sources, including experimental results, curated databases and computational predictions^60^. The database facilitates the exploration of functional associations between proteins, providing insights into the molecular networks that drive cellular processes. By compiling data from multiple sources, STRING offers a high-confidence representation of protein interactions, enabling researchers to construct robust protein interaction networks and better understand how proteins collaborate to maintain cellular function. The STRING database integrates information from a diverse range of experimental methods, including yeast two-hybrid screens, affinity purification followed by mass spectrometry, co-immunoprecipitation and other high-throughput techniques. In addition, STRING incorporates data from curated databases, such as those focused on biochemical pathways and molecular function, as well as computational predictions based on sequence homology, text mining and structural modeling.

#### BioGRID

BioGRID (version 4.4.198) is a comprehensive, curated repository that provides high-quality data on protein, genetic and chemical interactions across a wide range of organisms, including humans, model organisms and pathogens^61^. By consolidating diverse interaction data from various sources, BioGRID plays a role in supporting molecular biology, systems biology and biomedical research. The database is designed to facilitate the exploration of molecular networks, offering insights into how proteins, genes and chemicals interact to regulate cellular processes, maintain homeostasis and contribute to disease mechanisms. BioGRID curates data from a variety of experimental techniques, including yeast two-hybrid screens, co-immunoprecipitation, affinity purification–mass spectrometry and synthetic lethal screens. In addition to experimental data, BioGRID integrates information from computational predictions, enabling researchers to investigate both direct and indirect interactions within complex biological systems. This multisource approach ensures that the data within BioGRID are comprehensive, covering a wide range of biological interactions that help researchers gain a deeper understanding of cellular functions. The protein interaction data in BioGRID is valuable for constructing molecular interaction networks.

#### Gene regulation

Gene regulation databases play a critical role in understanding the complex mechanisms that control gene expression, including the processes that regulate the transcriptional, post-transcriptional and epigenetic aspects of gene activity (version 2.0)^62^. These databases integrate a wide range of information about how genes are turned on or off in response to various signals and how they maintain their expression patterns across different biological contexts. Specifically, these databases provide detailed insights into transcription factor binding, epigenetic modifications, noncoding RNA regulation and other molecular interactions that govern gene expression. One of the key components of gene regulation is the binding of transcription factors to specific DNA sequences within promoter regions or enhancers of target genes. Gene regulation databases, such as TRANSFAC and its module TRANSCompel, provide curated and experimentally validated data on transcription factor binding sites, offering researchers the ability to predict which transcription factors regulate specific genes.

#### CORUM

CORUM (version 3.0) is a comprehensive, curated resource dedicated to cataloging protein complexes in mammals, offering insights into the composition, function and interactions of these complexes that are essential for various cellular processes^63^. This extensive database serves as a key resource for understanding the molecular machinery underlying fundamental biological processes, including signal transduction, gene expression regulation, metabolism and cellular structure. By integrating experimental data from various sources, CORUM provides an up-to-date repository of information on the assembly, function and interactions of mammalian protein complexes. Protein complexes are groups of two or more proteins that interact to perform specific biological functions within the cell. The formation and regulation of these complexes are critical for maintaining cellular homeostasis, controlling signal transduction pathways and enabling cellular responses to internal and external stimuli.

#### National Library of Medicine

The National Library of Medicine hosts a range of biomedical and health-related resources, including the National Center for Biotechnology Information (NCBI). For the construction of the KG we incorporated gene and GO term associations derived from the NCBI Gene database (formerly known as Entrez Gene). Specifically, we used a processed dataset, which was based on the publicly available curated associations between genes and GO terms describing BP, MF and CC^4^. The associations (*n* = 297,917) between gene and GO terms from this dataset were incorporated as links in the KG.

### KG construction

Diverse biological databases were integrated to construct CardioKG. The data from various databases for each entity were harmonized by creating a dictionary that has all the possible ontologies of each node and then natural language processing library nltk was used to tokenize, clean and normalize the ontologies before matching with a reference list. For instance, the disease entity HF has various classifications such as congestive heart disease and left ventricular failure that required harmonization. The nodes in the constructed KG were extracted from 12 different entities including genes, diseases, medication, individuals, CMR, phenotypes, anatomy, BP, CC, MF, pathways and air pollutants, along with their relationships. The CMR features were incorporated into the KG as distinct nodes, by connecting them to heart anatomical regions such as left and right ventricle (Supplementary Fig. 3), with their corresponding values assigned as properties on the edges connecting the CMR nodes to other related nodes. In addition, individuals’ sex, age, BMI, ethnicity and BSA were integrated as properties within the corresponding individual nodes. Hence, the final graph has nodes (*N*), properties of nodes (*N*_p_), edges (*E*) and properties of edges (*E*_p_) as shown in equation (1)

1 $${\mathrm{KG}}=(N,E,{N}_{\mathrm{p}},{E}_{\mathrm{p}}),$$

### KG embeddings

In the KG, phenotypes, genes and diseases are represented as nodes connected by diverse relationships such as phenotype–gene, phenotype–pathway and gene–disease links. To capture both direct and indirect relationships, we generated embeddings that aggregate neighborhood information across multiple hops. This ensures that the embedding of a phenotype node reflects not only its immediate neighbors but also its indirect connectivity through intermediate nodes. Graph embedding techniques learn low-dimensional representations of nodes and edges while preserving the graph’s structural properties and relational patterns, enabling downstream tasks such as node clustering and link prediction. KG embedding methods, such as Node2Vec^15^ and ComplEx^17^, focus only on graph connectivity and fail to capture node and relation attributes. Therefore, DVGAE was employed as the embedding algorithm as it integrates both node and relation properties for generating embeddings. The DVGAE is a deep learning model that uses a variational graph auto-encoder^9^. However, our DVGAE networks extend traditional VGAE by handling edge direction, node type as well as node and relation properties in the KG (Supplementary Fig. 21). The components of the DVGAE are described below.

#### Encoder

The encoder uses multiple graph convolutional network (layers to process four inputs including node type, node properties, edge properties and edge direction to learn a low-dimensional latent representation). These embeddings are further enriched with node-type information to better represent the diversity of node roles in the graph. By learning these latent variables, the encoder enables the model to effectively capture the underlying relationships in the graph required for graph reconstruction and link prediction. The latent layer contains the low-dimensional embeddings generated by the encoder component of the directed variational graph auto-encoder, which captures the underlying structural and semantic information from the directed KG. These embeddings serve as compact representations of the nodes, preserving key relational patterns, and are used as input for downstream tasks.

#### Decoder

After the encoder generates the embeddings, the decoder takes these embeddings and predicts the presence or absence of edges between nodes. Specifically, the decoder calculates the dot product between pairs of node embeddings, which gives a measure of how strongly connected two nodes are. The final output is a set of probabilities indicating whether there should be an edge between each pair of nodes. The decoder thus enables the model to reconstruct the graph structure based on the learned node embeddings.

#### Validation

For graph-based learning tasks, it is essential for the model to capture the global structure of the graph, including relationships between distant nodes. To train the DVGAE model, the dataset was split into training (70%) and validation (30%) subsets. The embeddings produced by the encoder are used to compute the reconstruction loss and the Kullback–Leibler (KL) divergence loss, as defined in equations (2) and (3). The reconstruction loss evaluates the model’s ability to correctly predict edges, while the KL loss regularizes the latent space during training. Training is carried out using a grid search approach over a set of hyperparameters including learning rate {0.001, 0.01, 0.1}, number of epochs {50, 100, 200} and latent dimension sizes {25, 50, 100}. For each combination, the model is trained and evaluated on the validation set. The optimal configuration-learning rate of 0.001, 100 epochs, and a latent dimension size of 50 was selected based on the lowest loss. Validation loss is computed after each epoch by comparing the predicted edges to the true edges in the validation set. Negative edges are randomly sampled from node pairs that do not exist in the original graph, providing a contrastive signal for learning. The validation loss, defined in equation (4), serves as the basis for selecting the final model, with the configuration yielding the lowest validation loss chosen as optimal.

2 $${\mathcal{L}}_{\rm{recon}}=-\frac{\it{1}}{| {\mathcal{E}}^{+}| }\sum _{(u,v)\in {\mathcal{E}}^{+}}{\rm{log}} \left({\sigma} \left({\bf{z}}_{u}^{\top}{\bf{z}}_{v}\right)+{\varepsilon} \right),$$

where ${{\mathcal{E}}}^{+}$ denotes the set of positive (observed) edges, **z**_*u*_ and **z**_*v*_ are the learned embedding vectors for nodes *u* and *v*, *σ*(⋅) is the sigmoid function and *ε* is a small constant added for numerical stability.

3 $${\mathcal{L}}_{\rm{KL}}=-\frac{\it{1}}{\it{2}}\mathop{\sum }\limits_{j={\it{1}}}^{J}\left({\it{1}}+\log ({\sigma}_{j}^{\it{2}})-{\mu }_{j}^{\it{2}}-{\sigma }_{j}^{\it{2}}\right),$$

where *μ*_*j*_ and *σ*_*j*_ represent the *j*th dimension of the mean and s.d. vectors output by the encoder for a given node and *J* is the size of the latent space.

4 $$\it{{\mathcal{L}}}_{{\rm{total}}}={{\mathcal{L}}}_{{\rm{recon}}}+\frac{1}{N}\times {{\mathcal{L}}}_{{\rm{KL}}}.$$

The final DVGAE model, configured with the optimized hyperparameters, was used to generate node embeddings. To evaluate the quality of these embeddings, edge-wise accuracy (equation (5)) was employed as an evaluation metric. By comparing the reconstructed graph to the original graph on an edge-by-edge basis, this metric provides a quantitative assessment of how well the learned embeddings preserve the structural properties of the input graph.

5 $$\text{Edge-wise accuracy}=\frac{| \mathrm{TP}| }{| \text{original edge set}\,| },$$

where the true positive (TP) is the intersection of the original and reconstructed edge sets.

### Predicting potential gene–disease associations

#### HF

The embedding vector of the ‘heart failure’ node was concatenated with the embedding vectors of both positive and negative genes (Supplementary Table 3). Positive genes refer to those with a known association with HF, as identified from the standard database DisGeNET. Although DisGeNET was used in constructing the KG, only phenotype–gene and other nonevaluated associations were included, ensuring that no data leakage occurred during the predictive task. Negative genes, on the other hand, are those with no association with cardiovascular diseases, including HF, according to DisGeNET. The concatenated embeddings were assigned labels of either 0 or 1, as defined in equation (6) below.

6 $$\,\mathrm{label}\,=\left\{\begin{array}{ll}1,\quad &\,\text{if the gene is associated with HF}\,\\ 0,\quad &\,\text{if the gene is not associated with HF}\,\end{array}\right.$$

Subsequently, three machine learning algorithms—random forest (hyperparameters n-trees, max-features, max-depth, min-samples-split and min-samples-leaf), SVM (hyperparameters C, gamma and kernel) and ANNs (hyperparameters learning rate, dropout rate, batch size and epochs)—were trained on the concatenated embeddings to predict the association between the genes and disease using fivefold cross-validation. Each model was optimized using relevant hyperparameters: random forest (number of trees, maximum features, maximum depth, minimum samples for split and minimum samples per leaf), SVM (regularization parameter C, kernel coefficient gamma and kernel type) and ANN (learning rate, dropout rate, batch size and number of epochs). The performance of the classifiers was evaluated based on accuracy (as defined in equation (7)), AUC–ROC, sensitivity and specificity. Accuracy was used as a comparative metric between the three machine learning classifiers to identify the best-performing model, while the embeddings and probability scores produced by the models inherently capture multihop associations. The classifier demonstrating the best performance was then selected and used to predict potential associations between HF and genes that have unknown associations with HF. Each gene predicted to have a positive association with HF was assigned a probability generated by the machine learning model, which was then used to rank the predicted genes.

7 $$\begin{array}{l}{\mathrm{Accuracy}}=\frac{{\mathrm{TP}}+{\mathrm{TN}}}{{\mathrm{TP}}+{\mathrm{FP}}+{\mathrm{TN}}+{\mathrm{FN}}},\\{\mathrm{true}}\,{\mathrm{positives}}\,({\rm{TP}}),\,{\mathrm{true}}\,{\mathrm{negative}}\, ({\rm{TN}}),\\{\rm{false}}\, {\rm{positive}}\, ({\rm{FP}})\, {\rm{and}}\, {\rm{false}}\,{\rm{negative}} \,({\rm{FN}}).\end{array}$$

#### AF

The embedding of ‘atrial fibrillation’ node was concatenated with the embeddings of both positive and negative genes (Supplementary Table 7). Positive genes refer to those known to be associated with AF, while negative genes have no association with cardiovascular diseases, including AF. The same approach used for HF was then applied to AF to predict potential associations.

#### MI

In MI, embedding of ‘myocardial infarction’ node was concatenated with the embeddings of both positive and negative genes (Supplementary Table 5). Then the same approach used for HF and AF was applied to predict potential associations.

### Enrichment analysis

Enrichment analysis was performed for each disease separately to validate the predicted genes. In each disease, the top ten genes, ranked by their predicted probabilities from the machine learning classifier, were subjected to enrichment analysis. This analysis aimed to identify whether these predicted genes were associated with critical pathways relevant to each disease, including AF, HF and MI. The g:Profiler platform (https://biit.cs.ut.ee/gprofiler/gost) was utilized to perform the enrichment analysis, identifying significantly enriched pathways associated with the top ten predicted genes in each disease. The *P* values of the enriched pathways were adjusted using the Benjamini–Hochberg method to control for multiple testing. Subsequently, the enriched pathways were examined to determine whether they included relevant pathways associated with the disease. Hence, genes associated with pathways identified as critical for the disease were considered to be associated with the disease.

### Druggablity analysis

The druggability of the top ten predicted genes for each disease was evaluated using the DGIdb^14^ database, which catalogs known drug–gene interactions from multiple curated sources. DGIdb curates information on drugs that inhibit, activate or otherwise modulate the activity of specific genes or their protein products. Genes were considered ‘druggable’ if they had at least one reported interaction in DGIdb. This analysis aimed to identify which of the predicted genes protein could be effectively targeted and modulated by existing medications, providing insights into their potential as therapeutic targets. Five types of drug–gene interactions were considered in this analysis: agonist, inhibitor, modulator, activator and antibody. These were selected because they represent the most common and therapeutically relevant interaction types associated with druggable genes in prior studies and databases such as DGIdb^64^. While DGIdb includes additional interaction types (for example, binder), our focus was on those with clearer functional implications in gene regulation and therapeutic modulation.

### Assessing the importance of imaging data

The importance of the CMR features was assessed from two distinct perspectives including the structural characteristics of the graph and the influence on the predicted gene associations:

#### Structural characteristics of the graph

The CMR features were integrated as nodes in the KG, and to understand how influential they are the PageRank algorithm^22^ was applied to calculate scores for each node in the KG. PageRank is a centrality algorithm identifies highly influential nodes within the graph. It considers the entire structure of the graph, incorporating both direct and indirect relationships to quantify a node’s influence as a score. In biological networks, such as gene–disease association graphs, PageRank can highlight critical nodes that influence overall connectivity and information flow.

#### Influence on the predicted genes

To assess the influence of CMR features on the predicted gene–disease associations, the CMR nodes were excluded from the KG. The same methodology was then applied, including generating node embeddings, predicting gene–disease associations and conducting enrichment analysis. The results were compared to the scenario that included the CMR nodes to determine whether the predicted genes differed. In addition, the analysis examined whether the inclusion of CMR features led to the prediction of a greater number of pathways associated with the diseases of interest (AF, HF and MI). The number of identified critical pathways for each disease was calculated and employed to quantify the differences in predicted pathways between the two scenarios, then a chi-squared test was conducted to evaluate the difference between the two scenarios in terms of the number of identified pathways associated with the diseases.

### Minimal model configuration

To evaluate the minimal requirements of our framework, a minimal model configuration analysis was conducted by retaining only the core entity types considered essential for prediction: disease, gene, exposure, medication and anatomy. We excluded five entities with comparatively low PageRank scores (pathway, BP, CC, MF and phenotype) to determine and assess predictive performance. We then reran the KG-based prediction pipeline and compared the resulting top-ranked genes to those obtained from the full model.

### Drug repurposing

In drug repurposing, the same cohort was used (*n* = 9,584) and the embeddings representing the disease of interest were concatenated with the embeddings of medications that are either indicated or contraindicated in that disease as defined in equation (8) (Supplementary Tables 21–23). We used a ground truth of known medication–disease pairs from DrugBank^13^ database. Although DrugBank was incorporated in building the KG, only other nonassessed associations were included to prevent any data leakage during the predictive task. Three machine learning algorithms—SVM, random forests and ANN—were then trained and validated using this integrated data to predict potential associations between the disease of interest and previously unassociated medications. The validation of the top ten predicted medications (based on their assigned probabilities) was conducted in two phases. Initially, an enrichment analysis was performed on the drug targets to determine whether they were significantly associated with critical pathways related to the disease of interest. Subsequently, a survival analysis was carried out to assess the survival probability of individuals (with death as the outcome) with the disease who received the predicted medication. These individuals (*n* = 489) represent a subset of patients who were not part of the primary cohort of 9,584 and do not have imaging phenotype data, as such data were not required for this validation step. The survival functions were adjusted for age, sex, BMI and ethnicity. Validation of this survival analysis was conducted in the the BioVU repository. The endpoint was defined as ’adverse event’, which is the observation of either all-cause death or diagnosis of class 3 or 4 in the New York Heart Association classification system. To avoid biased estimates, propensity matching was applied, matching population of participants using any of the medications methotrexate, hydroxychloroquine and sulfasalazine versus those not using any of these. Populations were balanced for age, sex, ethnicity and presence of ischemic heart disease (ICD-10 I20–I25, ICD-9 410–414). A Cox proportional hazards model was run, correcting for age, sex and BMI. In addition, a graph-based validation was conducted by computing the cosine similarity (equation (9)) between the predicted medication and those currently used for the treatment or management of HF.

8 $$\mathrm{label}\,=\left\{\begin{array}{ll}1,\quad &\,\text{if the medication is indicated for the treatment}\\&\text{of the disease of interest}\\ 0,\quad &\,\text{if the medication is contraindicated for the treatment}\\&\text{of the disease of interest}\end{array}\right.$$

9 $$\begin{array}{l}\cos (\theta )=\frac{{\bf{A}}\times {\bf{B}}}{\parallel A\parallel \parallel B\parallel },{\mathrm{Embeddings}}\,{\mathrm{of}}\,{\mathrm{predicted}}\,{\mathrm{medication}}\,({\rm{A}})\\{\mathrm{and}}\,{\mathrm{embeddings}}\,{\mathrm{of}}\,{\mathrm{indicated}}\,{\mathrm{medications}}\,({\mathrm{B}}).\end{array}$$

### Independent validation of predictions

To evaluate whether common comorbidities influenced the results, we repeated the analysis by repeating the gene prediction after excluding patients with hypertension (*n* = 20), diabetes (*n* = 7), chronic kidney disease (*n* = 11) or chronic obstructive pulmonary disease (*n* = 15).

To assess the robustness of the predictions, we applied the same KG construction and machine learning pipeline to an independent set of participants from the UK Biobank who were not included in the original cohort. Predicted associations between genes, diseases and medications were compared between the original and validation cohorts to evaluate consistency of the top-ranked entities in HF.

### Statistics

All analyses were performed using Python (version 3.12.1) and Neo4j Desktop (version 5.20.0). Categorical variables were expressed as frequencies and percentages, while continuous variables were reported as medians with interquartile ranges. In g:Profiler, the hypergeometric test was used to calculate the *P* values for assessing the significance of the association between genes and pathways. Subsequently, the Benjamini–Hochberg method was applied to control the false discovery rate by adjusting the *P* values for multiple comparisons and a *P* value <0.05 was considered statistically significant. The chi-squared test was performed to assess the importance of the CMR features in terms of the number of identified pathways associated with the diseases. The survival functions were estimated based on fitted Cox models. Propensity matching for cardiovascular disease was performed using the nearest neighbour method in the R MatchIt package.

### Reporting summary

Further information on research design is available in the Nature Portfolio Reporting Summary linked to this article.

## Supplementary information

Supplementary Information Supplementary Figs. 1–21 and Tables 1–23.

Reporting Summary

Supplementary Data 1 Enrichment analysis results from gProfiler for AF.

Supplementary Data 2 Enrichment analysis results from gProfiler for HF.

Supplementary Data 3 Enrichment analysis results from gProfiler for MI.

Supplementary Table 4 Single Excel file that has all the supplementary tables.

## Source data

Source Data Fig. 1 The source data for Fig. 5, which shows the enrichment analysis for the repurposed drugs.

## Data Availability

Data from UK Biobank are available for approved research upon application from https://www.ukbiobank.ac.uk/enable-your-research/apply-for-access under the terms of the UK Biobank’s data access policy. Source data are provided with this paper.

## Code availability

The scripts for data analysis are publicly available via GitHub at https://github.com/ImperialCollegeLondon/cardioKG and via Zenodo at 10.5281/zenodo.16025952 (ref. ^65^).
