# Supplementary material for: A multimodal vision knowledge graph of cardiovascular disease
Source: Nat Cardiovasc Res. 2025 Dec 29;5(1):18–33. doi: 10.1038/s44161-025-00757-4 (PMC12811117; doi:10.1038/s44161-025-00757-4)
Supplement: Supplementary file 1 — Supplementary Figs. 1–21 and Tables 1–23. [file 44161_2025_757_MOESM1_ESM.pdf]

nature cardiovascular research

Supplementary information https://doi.org/10.1038/s44161-025-00757-4

A multimodal vision knowledge graph of cardiovascular disease

In the format provided by the authors and unedited

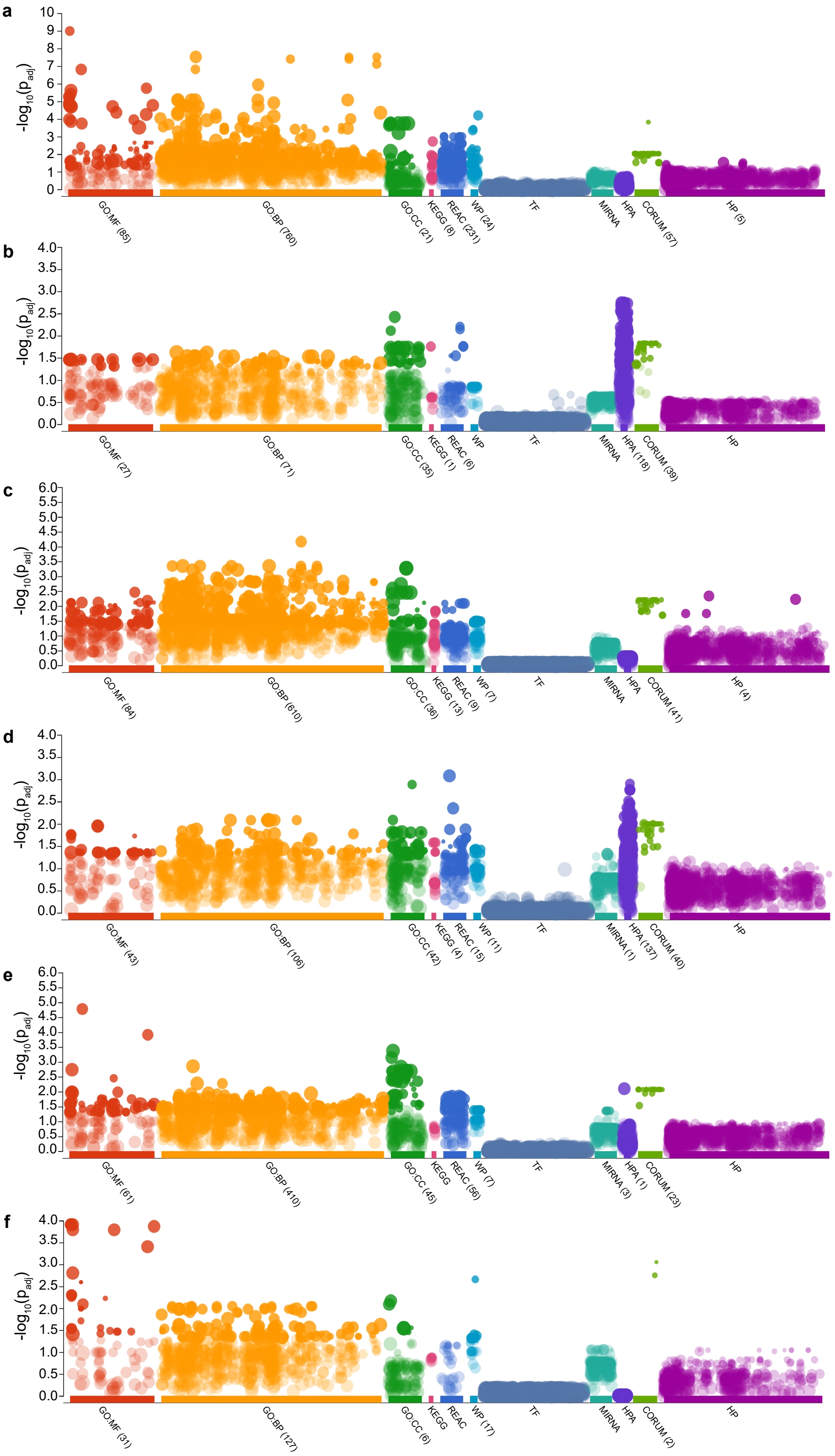

Supplementary Figure 1. The enrichment analysis for the top ten predicted genes in the diseases of interest. gprofiler has been used to perform the enrichment analysis and to generate the figures. x-axis represents the selected databases for the enrichment analysis and y-axis represents the adjusted p values using Benjamini-Hochberg (BH). **a:** is the enrichment analysis for heart failure (HF) using the predicted genes with integrating the cardiac magnetic resonance (CMR) data. **b:** is the enrichment analysis for HF using the predicted genes without the CMR. **c:** is the enrichment analysis for atrial fibrillation (AF) using the predicted genes with integrating the CMR. **d:** Shows the enrichment analysis for AF using the predicted genes without the CMR. **e:** is the enrichment analysis for myocardial infarction (MI) using the predicted genes with integrating the CMR. **f:** Shows the enrichment analysis for MI using the predicted genes without the CMR. GO: gene ontology, MF: molecular function, BP: biological process, CC: cellular component, KEGG: Kyoto Encyclopedia of Genes and Genomes, REAC: reactome, WP: WikiPathways, TF: TRANSFAC, MIRNA: miRTarBase, HPA: human protein atlas, HP: human phenotype.

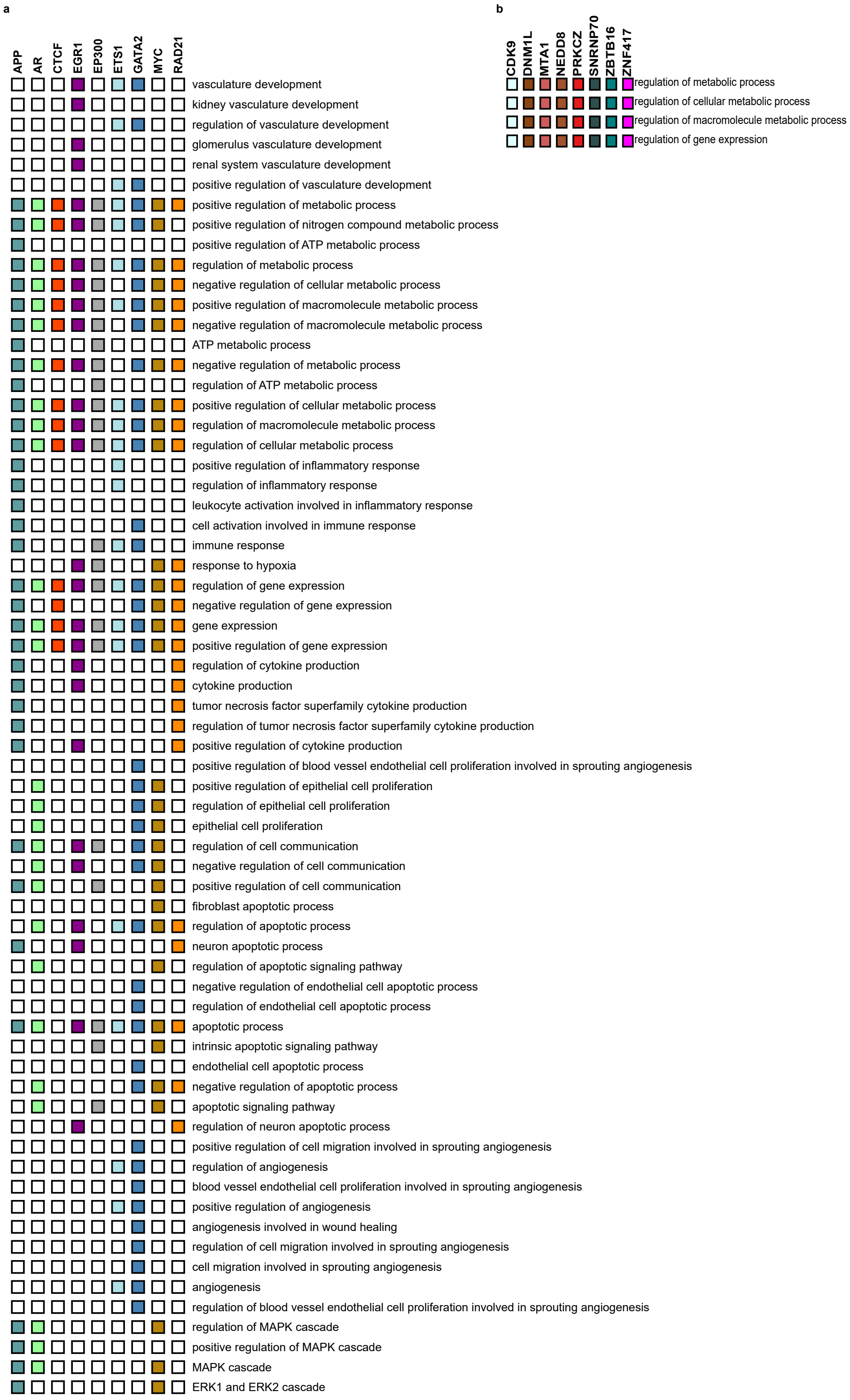

Supplementary Figure 2. The enriched pathways using the top predicted genes in heart failure. **a:** is the enriched critical pathways for heart failure (HF) using the predicted genes after integrating the CMR features in the knowledge graph (KG). Only the top nine predicted genes were significantly associated with critical pathways for HF. **b:** is the enriched critical pathways for HF using the predicted genes after excluding the CMR features from the KG. Only the top eight predicted genes were significantly associated with critical pathways for HF.

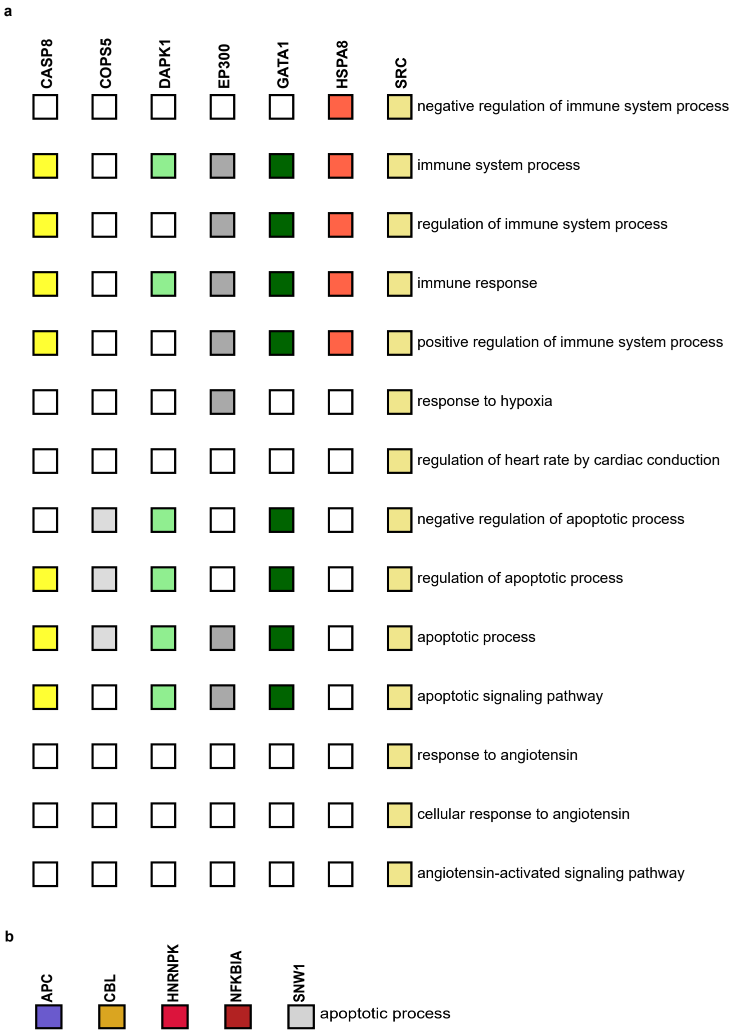

Supplementary Figure 3. The enriched pathways using the top predicted genes in atrial fibrillation. **a:** is the enriched critical pathways for atrial fibrillation (AF) using the predicted genes after integrating the CMR features in the knowledge graph (KG). Only the top seven predicted genes were significantly associated with critical pathways for AF. **b:** is the enriched critical pathway for atrial fibrillation using the predicted genes after excluding the CMR features from the KG. Only the top five predicted genes were significantly associated with critical pathways for AF.

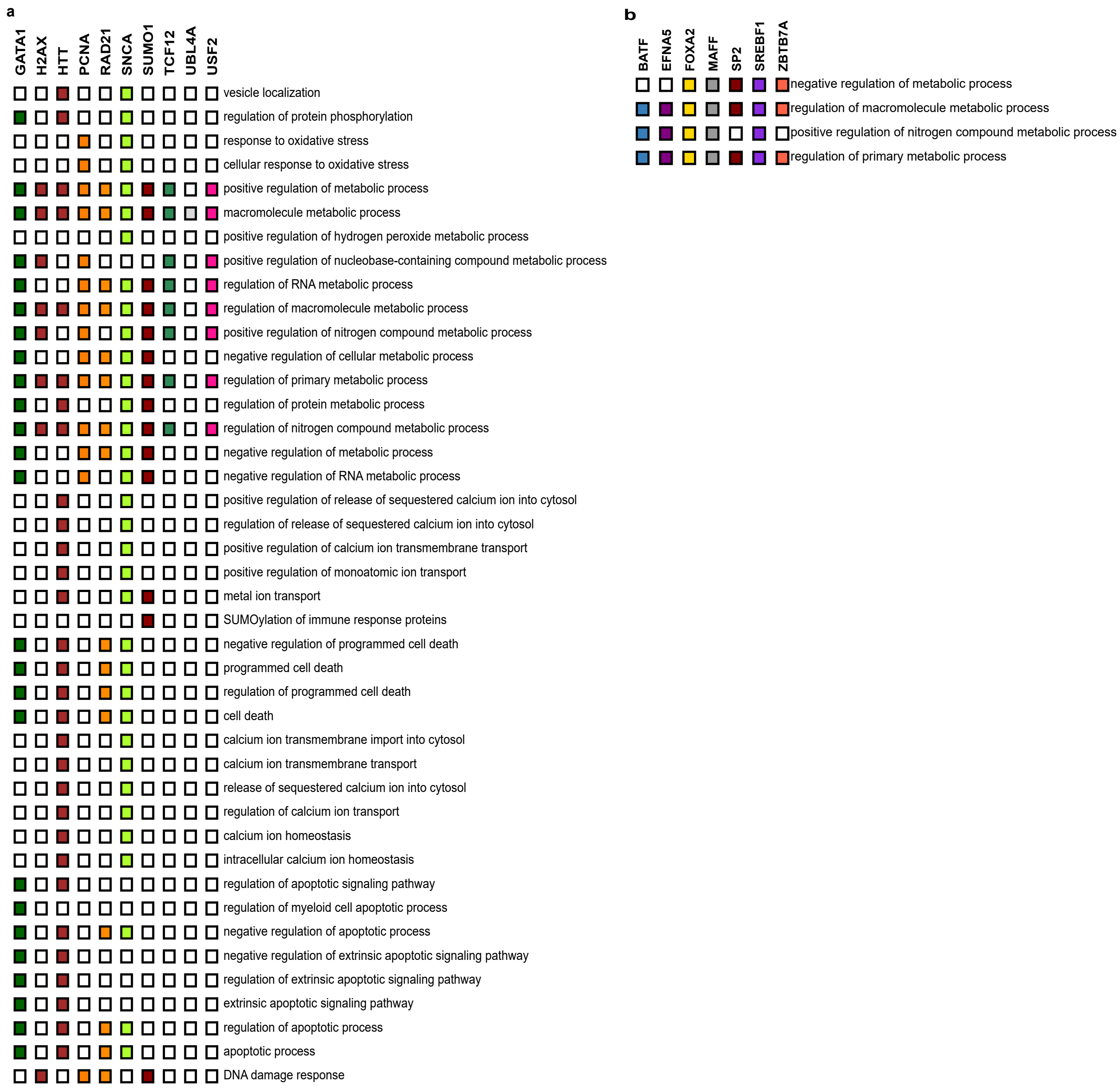

Supplementary Figure 4. The enriched pathways using the top predicted genes in myocardial infarction. **a:** is the enriched critical pathways for myocardial infarction (MI) using the predicted genes after integrating the CMR features in the knowledge graph (KG). The top ten predicted genes were significantly associated with critical pathways for MI. **b:** is the enriched critical pathway for myocardial infarction using the predicted genes after excluding the CMR features from the KG. The top seven predicted genes were significantly associated with critical pathways for MI.

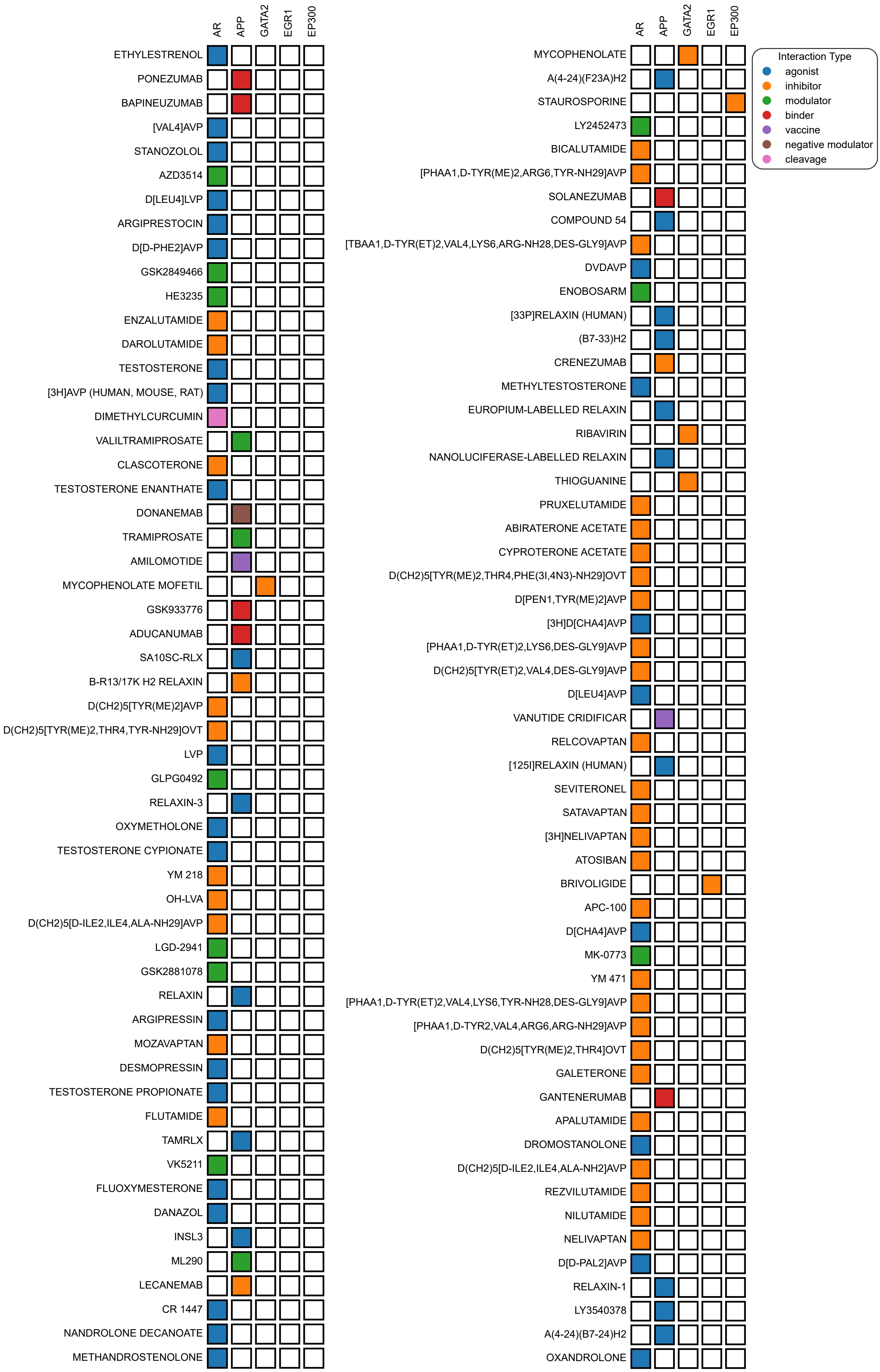

Supplementary Figure 5. The druggable genes in heart failure with CMR. Rows names represent medications and columns names represent genes.

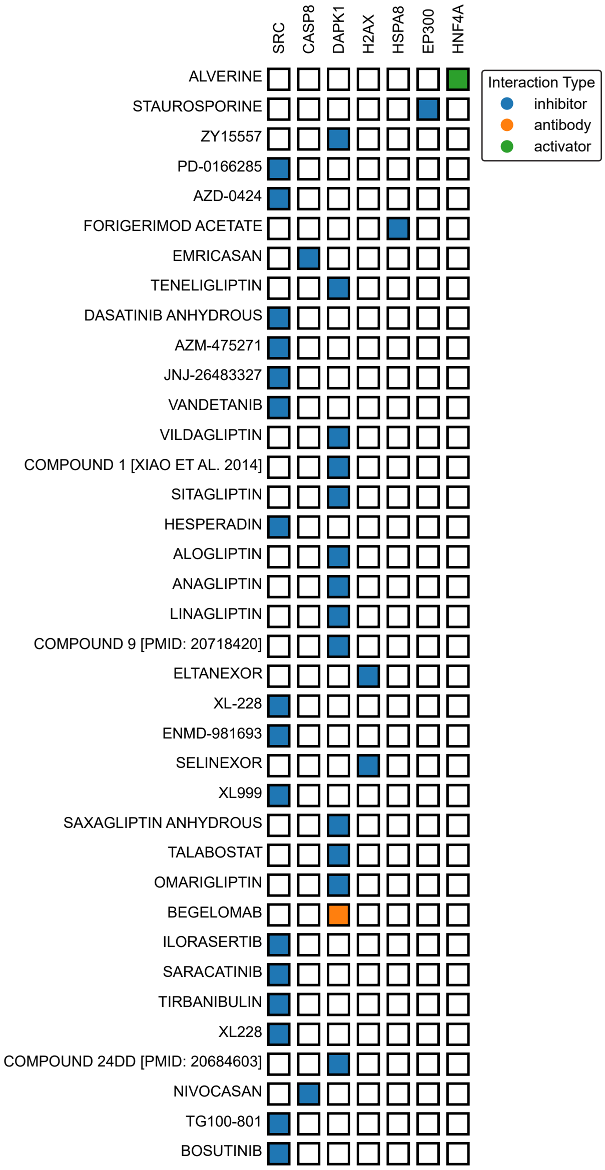

Supplementary Figure 6. The druggable genes in atrial fibrillation with CMR. Rows names represent medications and columns names represent genes.

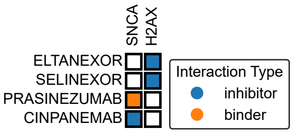

Supplementary Figure 7. The druggable genes in myocardial infarction with CMR. Rows names represent medications and columns names represent genes.

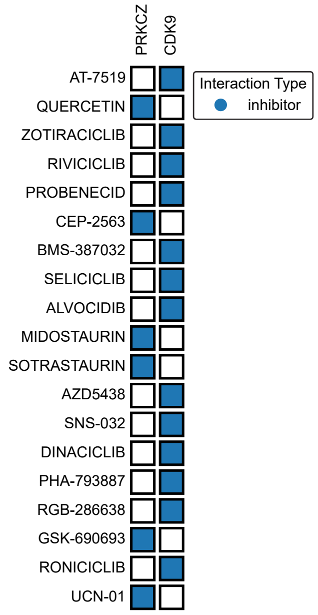

Supplementary Figure 8. The druggable genes in heart failure without CMR. Rows names represent medications and columns names represent genes.

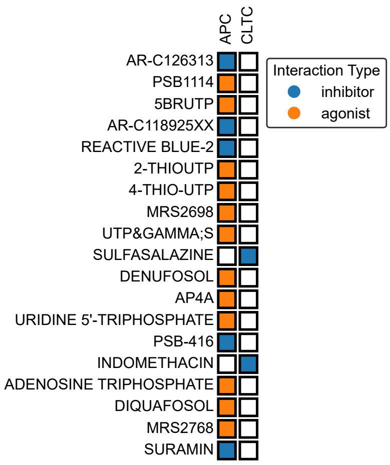

Supplementary Figure 9. The druggable genes in atrial fibrillation without CMR. Rows names represent medications and columns names represent genes.

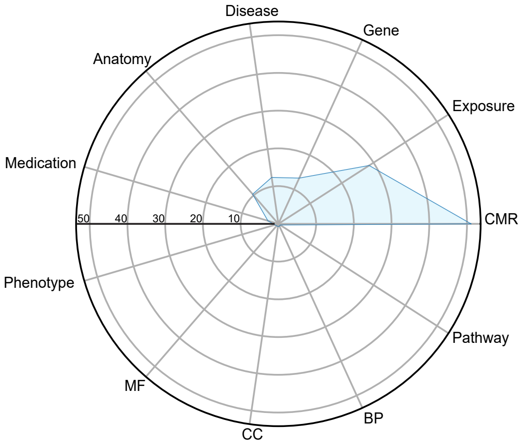

Supplementary Figure 10. The importance level of the entities in the knowledge graph (KG). The radar plot shows the PageRank scores for the entities in the KG. CMR: cardiac magnetic resonance, MF: molecular function, BP: biological process, CC: cellular component.

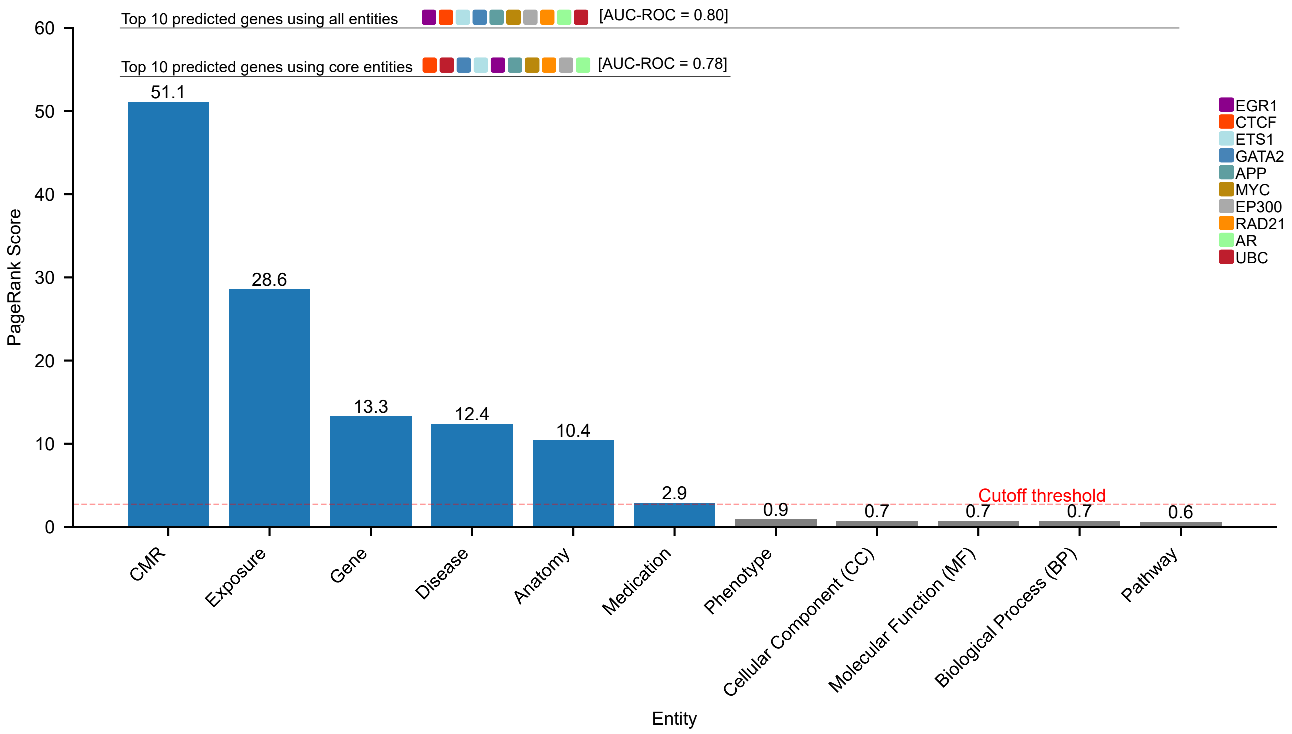

Supplementary Figure 11. Prediction performance across different entity configurations. The bar plot shows the PageRank scores for the entities in the knowledge graph (KG). The blue bars represent the core entities (minimal entities) that preserve the strong predictive performance for the model, while the gray bars show the entities that have a marginal impact on the predicted model and they can be excluded in scenarios where data availability is limited.

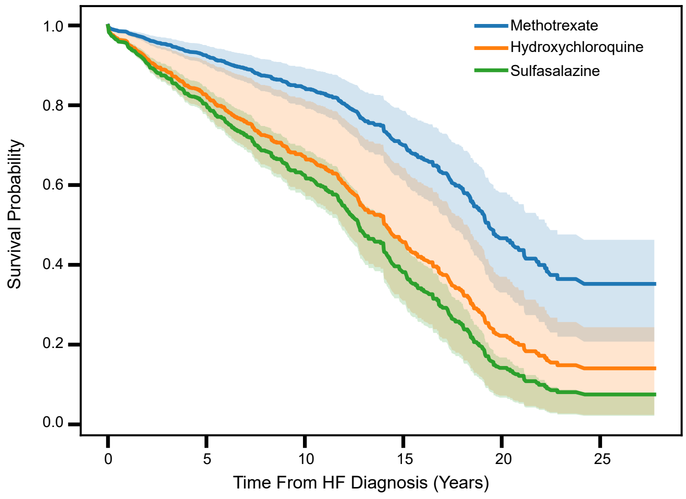

Supplementary Figure 12. Cox proportional hazards model–based survival curves for Methotrexate (n=121), Hydroxychloroquine (n=33), and Sulfasalazine (n=27). The baseline cohort consisted of patients diagnosed with both heart failure (HF) and rheumatoid arthritis (RA). Survival functions were adjusted for age, sex, BMI, and ethnicity. Shaded areas represent 95% confidence intervals. A statistically significant difference in survival was observed between the Methotrexate, Sulfasalazine and Hydroxychloroquine groups ($p < 0.01$).

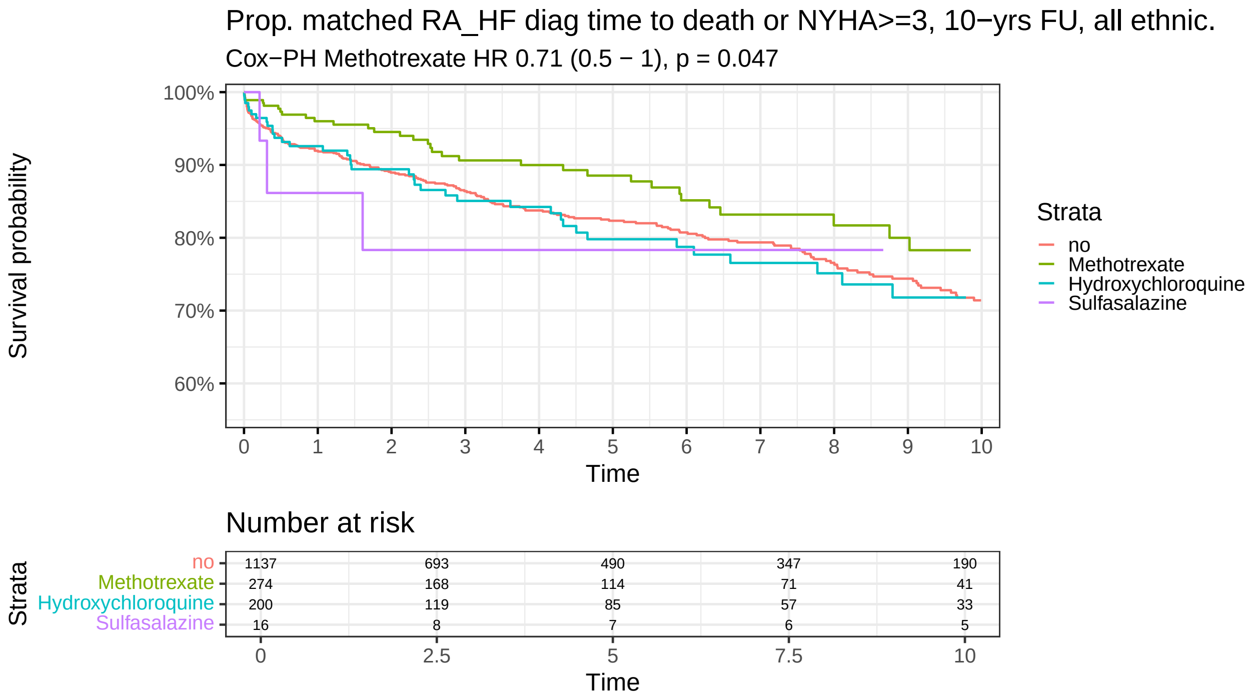

Supplementary Figure 13. Survival probability for adverse events under different medication in subjects with both Rheumatoid Arthritis (RA) and Heart Failure (HF) diagnoses. Endpoint 'adverse event' was defined as observing either all-cause death or NYHA class 3 or 4. HF diagnosis was defined via ICD10 in range I50 and ICD9 428. RA was defined as ICD10 M06 and ICD9 714. Methotrexate shows higher survival probability with an estimated hazard ratio of 0.7 in a corresponding CoxPH model. (Supplementary Table 12)

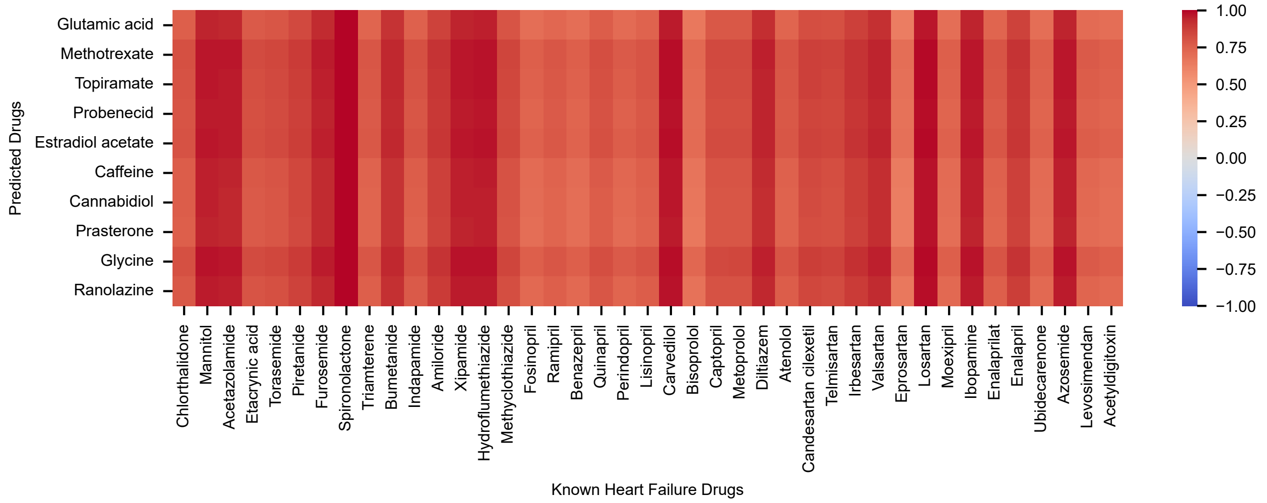

Supplementary Figure 14. Graph-based validation of the model-predicted medications for heart failure (HF) using embedding similarity analysis. The figure shows the cosine similarity scores between the predicted medications (based on the knowledge graph) and medications already indicated for HF in clinical practice. Higher cosine similarity indicates greater proximity in the embedding space, suggesting stronger functional or therapeutic relevance. Medications with higher similarity scores are more closely aligned with known HF treatments in the embedding space.

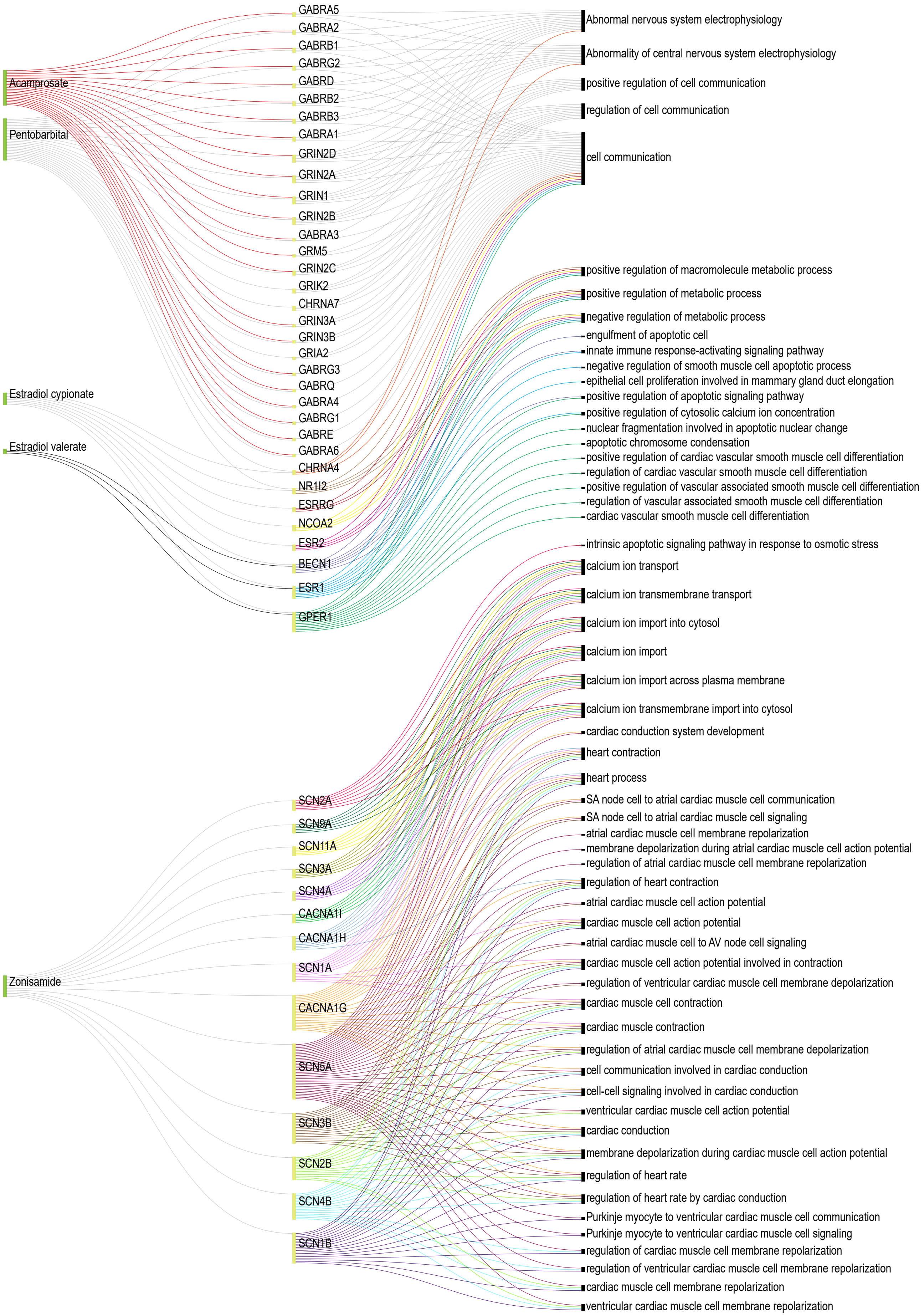

Supplementary Figure 15. Enrichment analysis results of target genes of predicted medications for atrial fibrillation. This figure displays the results for pharmaceutical compounds (n=5) only, excluding non-pharmaceutical substances (n=5).

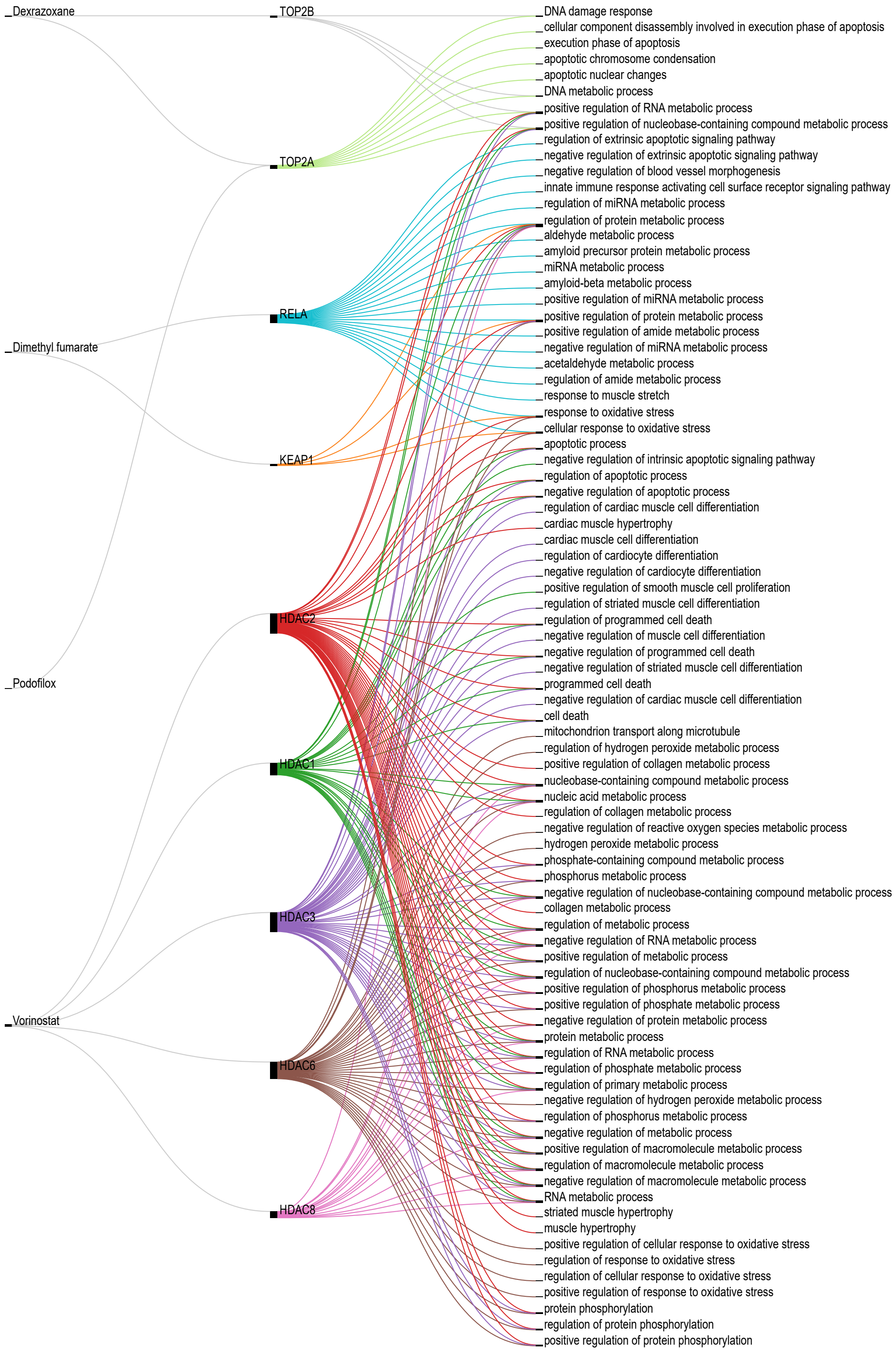

Supplementary Figure 16. Enrichment analysis results of target genes of predicted medications for myocardial infarction. This figure displays the results for the first four pharmaceutical compounds (n=4) only.

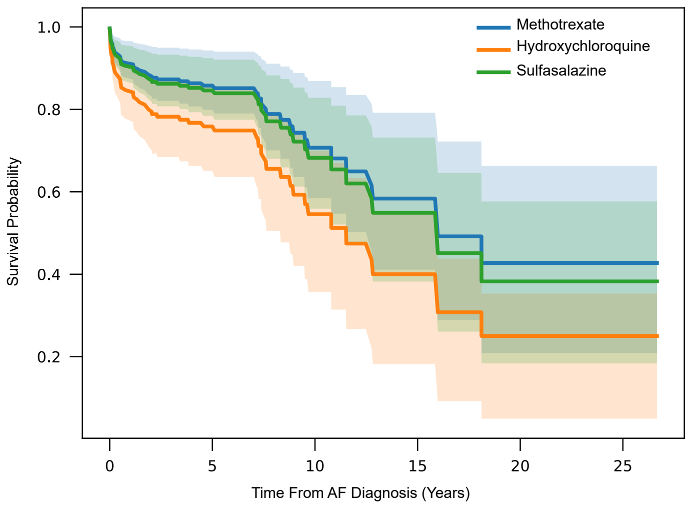

Supplementary Figure 17. Cox proportional hazards model–based survival curves for Methotrexate (n=170), Hydroxychloroquine (n=39), and Sulfasalazine (n=46). The baseline cohort consisted of patients diagnosed with both atrial fibrillation (AF) rheumatoid arthritis (RA). Survival functions were adjusted for age, sex, BMI, and ethnicity. Shaded areas represent 95% confidence intervals.

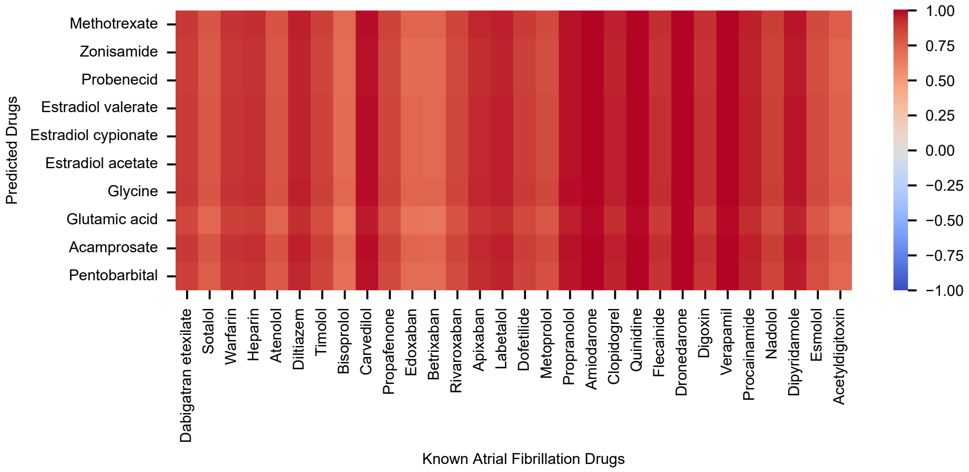

Supplementary Figure 18. Graph-based validation of the model-predicted medications for atrial fibrillation (AF) using embedding similarity analysis. The figure shows the cosine similarity scores between the predicted medications (based on the knowledge graph) and medications already indicated for AF in clinical practice. Higher cosine similarity indicates greater proximity in the embedding space, suggesting stronger functional or therapeutic relevance. Medications with higher similarity scores are more closely aligned with known AF treatments in the embedding space.

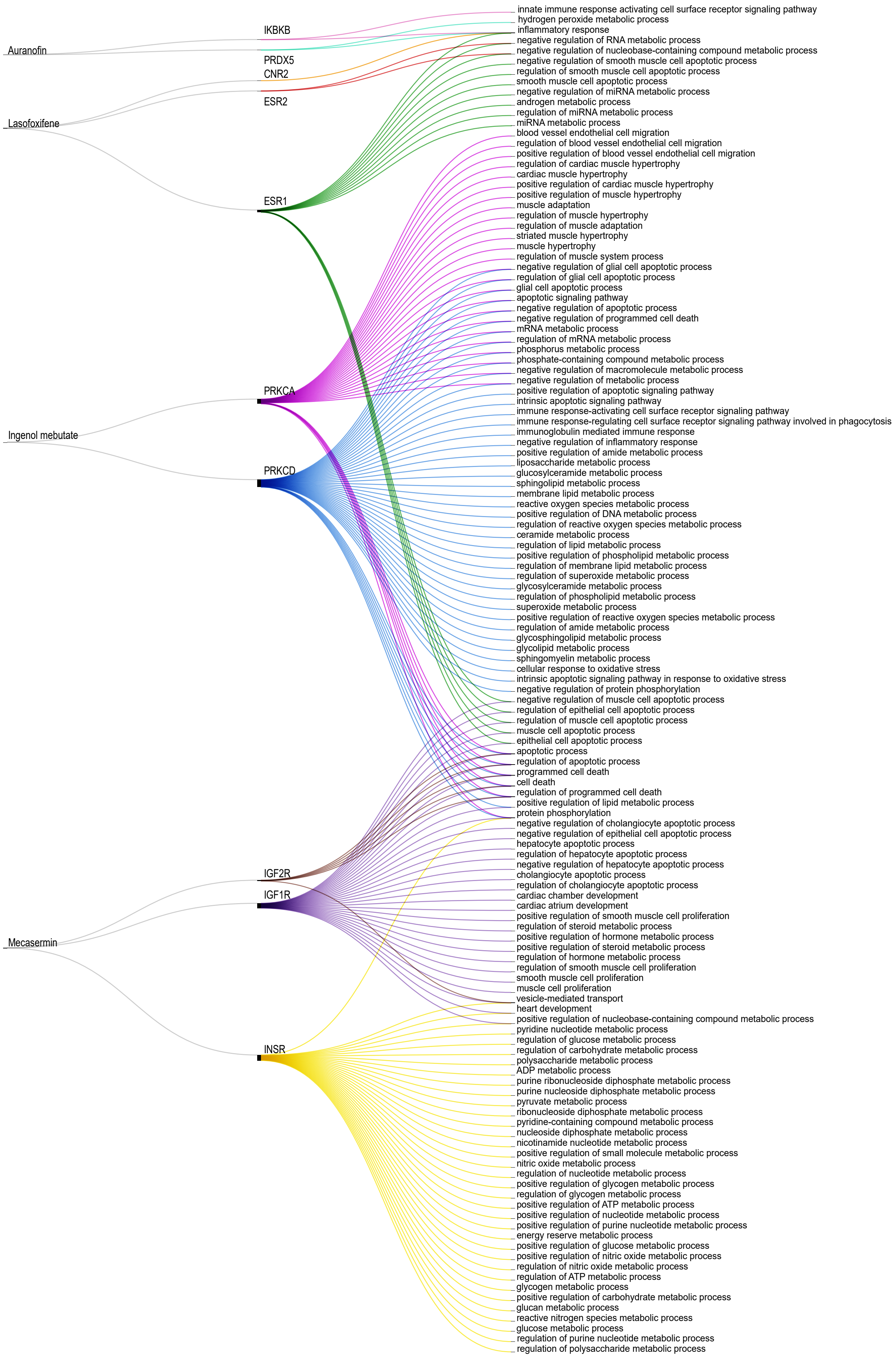

Supplementary Figure 19. Enrichment analysis results of target genes of predicted medications for myocardial infarction. This figure displays the results for the next four pharmaceutical compounds (n=4) only.

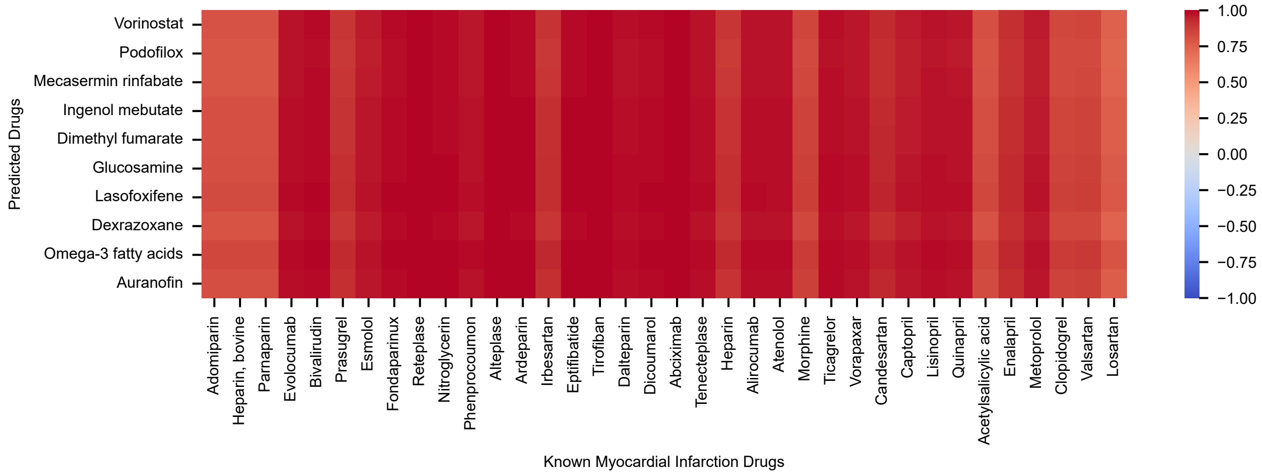

Supplementary Figure 20. Graph-based validation of the model-predicted medications for myocardial infarction (MI) using embedding similarity analysis. The figure shows the cosine similarity scores between the predicted medications (based on the knowledge graph) and medications already indicated for MI in clinical practice. Higher cosine similarity indicates greater proximity in the embedding space, suggesting stronger functional or therapeutic relevance. Medications with higher similarity scores are more closely aligned with known MI treatments in the embedding space.

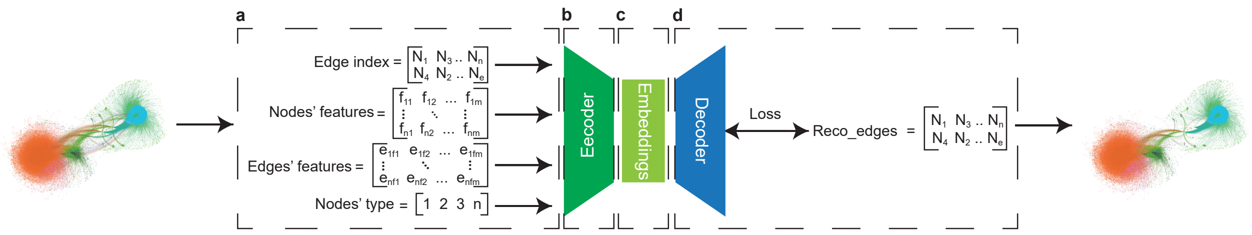

Supplementary Figure 21. The structure of the Embedding algorithm. The directed variational graph auto-encoder (DVGAE) operates on four key inputs: edge directions, node properties, relationship attributes, and node types. The encoder processes these inputs to generate embeddings in a latent space, while the decoder reconstructs the original graph to assess the embedding algorithm's performance and the quality of the generated embeddings. These latent-space embeddings are subsequently utilized for the downstream task of gene-disease association prediction. **a**: the key input (matrices and vectors). **b**: the encoder, which takes the graph as four key inputs. **c**: the latent space which has the generated embeddings from the encoder. **d**: the decoder which uses the embeddings to reconstruct the edges and evaluate the quality of the generated embeddings.

Supplementary Table 1. The used ICD-10 and ICD-9 codes to extract the disease. The codes that have been used to extract the individuals form the UK Biobank. ICD-10 is the tenth revision of the International Classification of Diseases, a system used by healthcare providers to code and classify diseases and health conditions. It replaced ICD-9.

Disease	ICD-10 code	ICD-9 code
Heart failure	I11.0, I13.0, I13.2, I50	428
Atrial fibrillation	I48.0, I48.1, I48.2, I48.3, I48.4, I48.9	4273
Myocardial infarction	I21, I22, I23	4109, 4129 410, 411, 412
Hypertrophic cardiomyopathy	I42.1, I42.2	4251
Dilated cardiomyopathy	I42.0, I42.6, I42.7	4254, 4255

Supplementary Table 2. The demographics and the imaging characteristics of the extracted cohort from the UK Biobank. All the individuals (n=9,584) have cardiac magnetic resonance imaging (CMR). Healthy includes individuals who have non of the following diseases: cardiovascular diseases, heart diseases, peripheral vascular disease, cerebrovascular diseases, hypertension, hyper-cholesterol, diabetes, cancer and chronic kidney disease. AF: atrial fibrillation, MI: myocardial infarction, HF: heart failure, HCM: hypertrophic cardiomyopathy, DCM: dilated cardiomyopathy, BMI: body mass index, BSA: body surface area.

**Baseline characteristics**	**Healthy** (n=5,304)	**AF** (n=2,153)	**MI** (n=1,414)	**HF** (n=602)	**HCM** (n=56)	**DCM** (n=55)
Sex Males(%)	1747(32.93%)	1494(69.39%)	1123 (79.42%)	441 (73.26%)	43 (76.79%)	45 (81.82%)
Ethnicity White(%)	4650 (87.67%)	2107 (97.86%)	1361 (96.25%)	588 (97.67%)	55 (98.21%)	54 (98.18%)
Age at time of MRI (years) Median[Q1;Q3]	61.0 [55.25; 67.0]	70.0 [65.0; 74.0]	69.0 [64.0; 73.0]	71.0 [66.0; 74.0]	67.0 [62.75; 71.0]	69.0 [63.0; 72.0]
Body Mass Index (kg/m^2) Median[Q1;Q3]	23.67 [21.83; 25.71]	26.31 [23.89; 29.39]	26.78 [24.34; 29.68]	26.67 [24.23; 30.1]	25.92 [24.19; 28.54]	26.83 [25.07; 28.83]
Body Surface Area (m^2) Median[Q1;Q3]	1.77 [1.65; 1.92]	1.97 [1.81; 2.12]	1.96 [1.82; 2.1]	1.97 [1.82; 2.13]	1.95 [1.79; 2.07]	2.04 [1.87; 2.13]
**CMR**	Median[Q1;Q3]	Median[Q1;Q3]	Median[Q1;Q3]	Median[Q1;Q3]	Median[Q1;Q3]	Median[Q1;Q3]
Left ventricle end systolic volume (ml)	31.07[26.62;36.41]	33.6[27.75;41.24]	34.77[28.48;43.15]	38.55[29.92;50.25]	35.47[28.54;42.46]	49.67[40.72;62.56]
Left ventricle end diastolic volume (ml)	78.57[70.4;87.33]	79.76[69.56;90.04]	81.85[71.76;93.8]	83.47[71.16;97.44]	79.54[72.3;92.18]	96.14[79.36;112.76]
Left ventricle systolic volume (ml)	47.13[41.91;52.97]	45.31[38.38;52.15]	46.21[40.27;51.93]	44.21[38.5;50.6]	45.86[41.3;52.71]	44.44[39.25;51.54]
Left ventricle cardiac output (ml)	5.05[4.36;5.86]	5.37[4.57;6.31]	5.2[4.51;6.07]	5.31[4.53;6.28]	5.44[4.61;6.12]	5.45[4.64;6.39]
Left ventricle ejection fraction (%)	60.08[56.44;63.76]	57.55[51.63;62.33]	57.06[51.48;61.68]	53.57[46.25;60.24]	57.53[51.43;63.45]	45.75[39.05;51.42]
Left ventricle mass (g)	42.54[38.03;48.4]	47.52[41.29;54.41]	49.7[43.84;55.29]	50.66[43.88;58.27]	56.93[48.13;71.86]	53.77[47.17;62.73]
Right ventricle end systolic volume (ml)	35.01[29.56;41.65]	37.6[31.26;45.73]	36.25[30.58;42.96]	37.82[31.19;46.62]	37.47[29.38;43.84]	44.42[38.3;52.27]
Right ventricle end diastolic volume (ml)	83.43[74.15;94.29]	83.33[73.01;94.72]	83.21[73.35;94.0]	82.5[72.15;93.73]	78.6[68.11;93.06]	82.73[76.71;95.09]
Right ventricle systolic volume (ml)	48.23[42.97;54.16]	45.63[38.14;53.03]	46.73[40.73;52.89]	43.85[38.24;50.86]	43.73[39.6;50.74]	39.9[36.59;45.55]
Right ventricle ejection fraction (%)	57.83[53.94;61.46]	55.01[48.9;59.76]	56.47[52.21;60.19]	53.96[48.34;59.28]	56.02[50.23;61.52]	48.54[41.68;53.37]
Left atrium maximum volume (ml)	37.93[31.54;44.96]	46.01[35.94;58.52]	40.41[32.55;49.8]	44.86[34.54;56.73]	44.86[37.99;62.56]	50.38[38.55;58.53]
Left atrium minimum volume (ml)	14.45[10.8;18.39]	21.67[14.62;35.72]	17.16[12.32;23.51]	20.87[13.83;31.89]	21.24[13.82;31.68]	23.39[17.63;37.82]
Right atrium maximum volume (ml)	46.98[39.79;55.64]	51.29[40.88;65.43]	44.18[36.44;54.34]	46.22[38.14;61.14]	47.83[36.1;62.0]	52.11[42.01;61.79]
Right atrium minimum volume (ml)	24.55[19.82;30.56]	28.88[21.23;41.02]	24.64[18.84;31.72]	26.36[19.62;36.89]	25.79[20.01;36.82]	31.47[22.89;40.16]
Descending aorta distensibility (10^{-3} .mmHg^{-1})	2.36[1.67;3.27]	1.79[1.33;2.47]	1.86[1.35;2.58]	1.7[1.22;2.44]	1.91[1.53;2.58]	1.85[1.37;2.56]
Ascending aorta distensibility (10^{-3} .mmHg^{-1})	1.69[1.12;2.56]	1.29[0.91;1.96]	1.32[0.94;1.94]	1.28[0.88;1.9]	1.33[0.97;1.72]	1.35[0.99;2.0]
Global longitudinal strain (%)	-18.56[-20.25;-16.99]	-17.21[-19.51;-14.55]	-17.67[-19.5;-15.65]	-16.62[-18.49;-13.95]	-16.64[-19.18;-14.86]	-15.72[-17.34;-11.47]
Circumferential peak diastolic strain rate (s^{-1})	2.53[2.06;3.03]	1.97[1.46;2.6]	1.95[1.46;2.5]	1.65[1.15;2.22]	1.95[1.36;2.62]	1.29[0.84;1.75]
Longitudinal peak diastolic strain rate (s^{-1})	1.75[1.4;2.14]	1.48[1.11;1.9]	1.46[1.14;1.86]	1.29[0.98;1.68]	1.33[1.13;1.95]	1.12[0.89;1.48]
Radial peak diastolic strain rate (s^{-1})	-6.23[-7.44;-5.07]	-5.17[-6.73;-3.81]	-5.09[-6.46;-3.75]	-4.31[-5.83;-2.99]	-5.0[-6.86;-3.49]	-3.02[-4.77;-2.09]
Maximum wall thickness (mm)	8.59[7.67;9.57]	10.25[9.13;11.41]	10.22[9.27;11.34]	10.39[9.22;11.6]	12.45[11.17;15.21]	10.43[9.15;12.05]

Supplementary Table 3. Positive and negative genes for HF. The positive (n=143) and negative (n=143) genes were identified from the DISGENET database. HF: heart failure.

Positive genes for HF	Negative genes for HF
'PIK3IP1', 'SERPINE1', 'IL6', 'FIBIN', 'EDNRA', 'KAT8', 'ADRA1D', 'PTH', 'ALOX15', 'SLC9A1','PTGS1', 'IFNG', 'CIDEA', 'UCP1', 'ATP2A1', 'NPPA', 'MYH6', 'TNFRSF1A', 'CYRIB', 'WDR45', 'APLN', 'NRG1', 'MS4A6A', 'NOS2', 'MSTN', 'SIRT1', 'PTPN3', 'VEGFA', 'ALB', 'PTP4A2', 'CXCL2','BAMBI', 'INS', 'APCS', 'EPN3', 'EDNRB', 'ATP2A2', 'RAC1', 'HSPB1', 'ACACA', 'CFD', 'POMC', 'PTGS2', 'ROCK2', 'NR3C2', 'GHRL', 'SOD1', 'PON1', 'AGTR1', 'PDPK1', 'PIK3CG', 'ADIPOQ', 'PLAT', 'EPHX2', 'ADRB3', 'HMOX1', 'PCK1', 'TNF', 'TRDN', 'FASN', 'CXCL8', 'NPPB', 'AVP', 'NUPR1', 'SFRP1', 'CSF2', 'HAND2', 'AGT', 'PDGFRA', 'TLR2', 'APOC1', 'FIP1L1', 'FXYD3','ITGB1', 'MYH7', 'HAMP', 'REN', 'HTR2B', 'PPP1R1A', 'PEBP1', 'CRP', 'NPR1', 'GCG', 'SOD3', 'CSF3', 'CCN2', 'COL4A1', 'RETN', 'PLXND1', 'PPARG', 'NRIP1', 'FBLN5', 'NOX4', 'COL6A1', 'SOX4', 'CREG1', 'ADRB1', 'ACADS', 'CAT', 'ELOVL6', 'AKIP1', 'CYBB', 'NFE2L2', 'PRL', 'ADRA2C', 'NOS3', 'XDH','CCL2', 'ANKRD23', 'EDN1', 'COL8A1', 'GRK2', 'TNNT2', 'ATP1A3', 'GSK3B', 'PPARGC1A', 'ACLY', 'AVPR2', 'ACE', 'KANK2', 'CLIC2', 'VWF', 'PRKAR2B', 'CDC25B', 'FHL1', 'TBX20', 'CS', 'GPX4', 'SOD2', 'RBP4', 'IL1B', 'OLR1', 'DSTN', 'SIPA1L1', 'MAP2K7', 'KLF9', 'GDF15', 'CXCL3', 'UCN2','NOX1', 'ELK3', 'GATM', 'HIF1A'	'HABP2', 'CRACR2B', 'RBMY2BP', 'HINT1', 'PSG2', 'GPT', 'ADAMDEC1', 'IGKV1D-39', 'NBAS', 'PRDM12','AAGAB', 'POC1B-GALNT4', 'GID4', 'OPA3', 'TEP1', 'UPP1', 'GPHA2', 'HINT3', 'BTF3L4', 'DNAL4','NSMCE1', 'EP400P1', 'LRRC8E', 'FAM43A', 'PEX13', 'OR2H1', 'PAGR1', 'CORT', 'NSA2', 'ADAMTS4', 'VWA8', 'FGF23', 'OPN1LW', 'MGST1', 'S1PR2', 'TAS2R13', 'WFDC9', 'SPTLC3', 'MAMDC2', 'PAPOLG', 'SWAP70', 'NR2F1', 'CHTF8', 'KLHL10', 'KCNMB2', 'BCLAF3', 'UNC79', 'DLGAP3', 'RNF115', 'EMSY','SLC15A3', 'CNPY1', 'WASH6P', 'CATSPERD', 'ESCO2', 'METTL27', 'ITFG2', 'SETD5', 'ARMC12', 'ST14','APCDD1L', 'SCARNA27', 'ANKRD53', 'ZNF350', 'SLC25A31', 'GALNT6', 'WFDC2', 'IGHV4-31', 'SMIM2', 'ETHE1', 'QRICH2', 'PPFIBP2', 'PCF11', 'ALDH5A1', 'SACS', 'HRC', 'C12orf29', 'C9orf153', 'SIKE1','COX6A2', 'KLHL21', 'FAM131A', 'TAS2R7', 'HOATZ', 'CRYGS', 'RFLNA', 'GMEB1', 'CYP46A1', 'MIR425', 'SMIM19', 'TPTEP1', 'METTL17', 'MED12L', 'SERF1A', 'AGPAT5', 'TMEM54', 'PATE1', 'KLHL3', 'C3orf35', 'ABTB1', 'RPS6KC1', 'ABCB7', 'C16orf46', 'SPATA13', 'ZNF347', 'STEAP4', 'PCDH9', 'FREM1', 'AHSA1','SPATA1', 'SOWAHC', 'IGLV3-19', 'IL17RD', 'GRSF1', 'CABCOCO1', 'ABR', 'ROBO1', 'TLR7', 'SH3BGR','CYP27C1', 'KIF3B', 'H2AZ2', 'ESRRB', 'LAD1', 'PSORS1C3', 'ASCL4', 'TMIGD3', 'OSBPL7', 'CLP1', 'HES5', 'SELENOM', 'IGLV3-12', 'VAX2', 'ARMC2', 'FECH', 'GSTT2', 'TCN1', 'PDZD11', 'ALDOB','FAM72A', 'GAGE5', 'ENAH', 'IGKV4-1'

Supplementary Table 4. Positive and negative genes for AF. The positive (n=160) and negative (n=160) genes were identified from the DISGENET database. AF: atrial fibrillation.

Positive genes for AF	Negative genes for AF
’GATA4’,’UBE4B’,’SCN10A’,’PPP2R3A’,’UST’,’AKAP6’,	SPATA13’, ’PRDM12’, ’VWA8’, ’PAGR1’, ’S1PR2’, ’CHAMP1’,
’LHX3’,’CEP68’,’LINC00477’,’WNT3’,’PPFIA4’,	’WFDC9’, ’EP400P1’, ’FAM131A’, ’KLHL3’,
’PRRX1’,’WDR1’,’MBD5’,’IRF2BPL’,’SYNPO2L’,	’AHSA1’, ’KCNMB2’, ’SLC25A31’, ’SIKE1’,’DUOXA1’, ’CHTF8’,
’MYPN’,’TBX5’,’XPO1’,’HAND2’,’KCNN2’,’GOPC’,	’GSTT2’, ’OSBPL7’, ’PCF11’, ’TLR7’,’AAGAB’, ’MGST1’,
’TEX41’,’CASQ2’,’CAV2’,’NPPB’,’RPL3L’,’KCNN3’,	’MAMDC2’, ’DNAL4’, ’VAX2’, ’IGKV4-1’, ’CLP1’, ’UNC79’, ’CYP46A1’,
’SSPN’,’SPATS2L’,’PITX2’,’NEURL1’,’GCOM1’,’VWF’,	’CRYGS’,’CRACR2B’, ’ZNF347’, ’NR2F1’, ’ADAMTS4’, ’ISCU’,
’LRIG1’,’KDM1B’,’PHLDB2’,’PLN’,’TNFSF12’,’SLC9B1’,	’FAM72A’, ’AGPAT5’, ’KIF3B’, ’CABCOCO1’, ’ARMC2’,’SMIM2’,
’NKX25’,’KIF3C’,’KCNJ8’,’RPS2’,’CAV1’,’ARHGAP10’,	’CALCOCO1’, ’ETHE1’,’HRC’, ’PEX13’, ’SH3BGR’, ’NFASC’,
’HIP1R’,’HCN4’,’PKP2’,’IGF1R’,’ABCC9’,’SOX5’,	’CYP27C1’, ’MED12L’, ’NBAS’,’SOWAHC’, ’ALDOB’, ’ABTB1’,
’DNAH10’,’KCNE5’,’LRMDA’,’SMAD7’,’SCMH1’,’TTN’,	’TMIGD3’, ’FREM1’, ’BBIP1’, ’PATE1’, ’GMEB1’,
’HSF2’,’MYH6’,’ATXN1’,’CREB5’,’MYOCD’,’PSMB7’,	’OPA3’, ’TAS2R13’, ’ABR’,’C5orf34’, ’ARMC12’, ’RFLNA’,
’ARNT2’,’ASAH1’,’TUBA8’,’NR3C1’,’AGBL4’,’AOPEP’,	’TCN1’, ’PDZD11’, ’SWAP70’, ’TEP1’, ’COX6A2’, ’HINT1’,
’NACA’,’DGKB’,’MYOT’,’SGCA’,’CYTH1’,	’SCARNA27’,’HINT3’, ’TTC8’, ’PSG2’, ’DHRS9’, ’ITFG2’,
’REEP1’,’CAND2’,’MYH7’,’SYNE2’,’GTF2I’,’SH3PXD2A’,	’HABP2’, ’USB1’, ’NSMCE1’, ’TAS2R7’, ’SLITRK4’, ’CNPY1’,
’COG5’,’RBM20’,’MYO1C’,’HERC1’,’LINC01426’,’CASZ1’,	’C9orf153’, ’IGHV4-31’, ’POC1B-GALNT4’, ’METTL17’, ’TUBD1’, ’ESCO2’,
’FBRSL1’,’WNT8A’,’MYOZ1’,’WIPF1’,’CUL4A’,’CGA’,	’TMEM54’, ’ANKRD53’,’FECH’,’IGLV3-12’, ’CORT’, ’GPHA2’,
’SLC35F1’,’CDKN1A’,’SCN5A’,’USP3’,’C1orf185’,’XPO7’,	’C12orf29’, ’MED11’, ’BTF3L4’,’STEAP4’, ’MIR425’, ’C16orf46’,
’PHLDA1’,’KCNJ5’,’ZPBP2’,’GNB4’,’OPN1SW’,’XXYLT1’,	’WNT3A’,’C3orf35’, ’TPTEP1’, ’LAD1’, ’SETD5’, ’OR2H1’,
’USP34’,’ZFHX3’,’MIR11HG’,’ARMH3’,’FGF5’,’FRMD4B’,	’PAXX’, ’GAGE5’, ’GID4’, ’ZNF350’, ’PSORS1C3’,
’SORL1’,’CYP2C19’,’NOS3’,’CDK6’,’HSPG2’,’LRRC10’,	’IGKV1D-39’, ’RPS6KC1’, ’NSA2’, ’RBMY2BP’,’ROBO1’, ’METTL27’,
’LINC01142’,’HTR4’,’ERBB4’,’SLIT3’,’THRB’,’MIR6500’,	’SMIM19’, ’ASCL4’, ’HOATZ’,’APCDD1L’, ’KLHL21’, ’SPATA1’
’KCNA5’,’GOSR2’,’SLC27A6’,’SNRNP27’,’KCNH2’,’KCNE2’,	, ’FAM43A’,’GALNT6’, ’GRSF1’, ’ST14’, ’DLGAP3’, ’FGF23’,
’NUCKS1’,’KCNJ2’,’PTK2’,’PAK2’,	’GPT’, ’QRICH2’,’SACS’, ’ADAMDEC1’, ’ALDH5A1’, ’IGLV3-19’,
’SIRT1’,’DPF3’,’SOX15’,’ZNF462’,’SUN1’,’CFL2’,	’UPP1’, ’ABCB7’, ’GLP2R’, ’SELENOM’, ’ESRRB’, ’WASH6P’,
’SELE’,’CAMK2D’,’ACE’,’DES’,’REEP3’,’BEST3’,	’OPN1LW’, ’KLHL10’, ’IL17RD’, ’HES5’,’WFDC2’,’PCDH9’,
’CALU’,’FBXO32’,’EPHA3’,’MYO18B’,’NEBL’,’MAPT’,	’AK8’,’EMSY’, ’ENAH’, ’SERF1A’, ’H2AZ2’, ’PPFIBP2’,
’KCND3’,’GYPC’,’ESR2’,’EDN1’,’TTNAS1’,’MIR30B’,	’RNF115’,’SLC15A3’,’CATSPERD’, ’SPTLC3’, ’LRRC8E’, ’PAPOLG’,
’NAV2’,’MEX3C’,’GJA5’	’BCLAF3’

Supplementary Table 5. Positive and negative genes for MI. The positive (n=95) and negative (n=90) genes were identified from the DISGENET database. MI: myocardial infarction.

Positive genes for MI	Negative genes for MI
’LDHA’, ’MIR145’, ’NR3C2’, ’NPPA’, ’TNNI3’,’AGT’,	’SPATA13’, ’PRDM12’, ’VWA8’, ’PAGR1’, ’S1PR2’, ’CHAMP1’,
’PPARGC1A’, ’EPO’, ’MYH7’, ’BRAP’, ’PDE3A’, ’SH2B3’,	’WFDC9’, ’EP400P1’, ’FAM131A’, ’KLHL3’,’AHSA1’, ’KCNMB2’,
’S100B’, ’BAK1’, ’SOD2’, ’KLF13’, ’BECN1’, ’ADORA1’,	’SLC25A31’, ’SIKE1’, ’DUOXA1’, ’CHTF8’, ’GSTT2’, ’OSBPL7’,
’F13A1’, ’DGKZ’, ’MIR125B2’, ’BCL2’,’APOE’, ’TGFB1’,	’PCF11’, ’TLR7’,’AAGAB’, ’MGST1’, ’MAMDC2’, ’DNAL4’, ’VAX2’,
’NOS3’, ’P2RY12’, ’ATM’, ’MFF’, ’GGT1’, ’LRP8’, ’MMP2’,	’IGKV4-1’, ’CLP1’, ’UNC79’, ’CYP46A1’, ’CRYGS’,’CRACR2B’,
’TM6SF2’,’PHACTR1’, ’F2’, ’GSK3B’, ’WDR12’, ’NOS2’,	’ZNF347’, ’NR2F1’, ’ADAMTS4’, ’ISCU’, ’FAM72A’, ’AGPAT5’,
’ACTA2’, ’BCL2L1’, ’ICAM1’, ’PSMA6’, ’MMP9’,’ADORA3’,	’KIF3B’, ’CABCOCO1’, ’ARMC2’, ’SMIM2’, ’CALCOCO1’,
’DDIT3’,’REN’, ’SOD1’, ’PLAT’, ’LGALS3’, ’IL1RN’, ’IL1B’,	’ETHE1’, ’HRC’, ’PEX13’, ’SH3BGR’, ’NFASC’, ’CYP27C1’,
’ITGB3’, ’HSPA5’,’PLAU’, ’SELL’, ’CASP3’, ’F5’, ’PAPPA’,	’MED12L’, ’NBAS’,’SOWAHC’, ’ALDOB’, ’ABTB1’, ’TMIGD3’,
’NRIP1’, ’ESR1’, ’THBD’,’LTA’, ’CKM’, ’HP’,’F7’, ’ATP1A1’,	’FREM1’, ’BBIP1’, ’PATE1’, ’GMEB1’, ’OPA3’, ’TAS2R13’, ’ABR’,
’CKB’, ’CAT’, ’MIA3’,’PRKCE’, ’GATA4’, ’GCLM’,’KLK1’,	’C5orf34’, ’ARMC12’, ’RFLNA’, ’TCN1’, ’PDZD11’, ’SWAP70’,
’HMGB1’, ’KIF6’, ’HSD11B2’,’MIR423’, ’TNF’, ’IL10’,	’TEP1’, ’COX6A2’, ’HINT1’, ’SCARNA27’,’HINT3’, ’TTC8’,
’OLR1’, ’LGALS2’, ’MIR761’, ’IFNA2’,’BAX’, ’ACE’,	’PSG2’, ’DHRS9’, ’ITFG2’, ’HABP2’, ’USB1’, ’NSMCE1’, ’TAS2R7’,
’MIAT’, ’GCLC’, ’CREB1’, ’CREM’,’PLN’, ’TNFSF4’	’SLITRK4’, ’CNPY1’,’C9orf153’, ’IGHV4-31’, ’POC1B-GALNT4’,
	’METTL17’, ’TUBD1’, ’ESCO2’, ’TMEM54’, ’ANKRD53’

Supplementary Table 6. The performance of the ML classifiers in predicting gene-disease associations. The accuracy (Acc) and area under the curve (AUC) for predicting the links between the genes and the diseases using three machine learning classifiers including random forest (RF), support vector machine (SVM) and artificial neural networks (ANNs). CMR: cardiac magnetic resonance imaging, AF: atrial fibrillation, MI: myocardial infarction, HF: heart failure.

Disease	Classifier	CMR		NO CMR	
		Acc	AUC	Acc	AUC
HF	SVM	72.4%	0.80	70.6%	0.79
	RF	62.1%	0.72	65.5%	0.70
	ANN	69.0%	0.80	70.6%	0.79
AF	SVM	75.0%	0.78	75.0%	0.76
	RF	71.9%	0.75	70.3%	0.75
	ANN	73.4%	0.78	68.3%	0.78
MI	SVM	83.3%	0.83	81.1%	0.82
	RF	75.6%	0.79	81.0%	0.82
	ANN	83.7%	0.82	70.2%	0.83

Supplementary Table 7. Top 10 predicted genes and their probabilities for each disease with and without CMR features. CMR:cardiac magnetic resonance imaging, HF: heart failure, AF: atrial fibrillation, MI: myocardial infarction.

HF				AF				MI			
CMR		NO-CMR		CMR		NO-CMR		CMR		NO-CMR	
Gene	Probability	Gene	Probability	Gene	Probability	Gene	Probability	Gene	Probability	Gene	Probability
EGR1	0.99	PRKCZ	0.98	DAPK1	0.99	CLTC	0.99	SUMO1	0.99	MAFF	0.99
CTCF	0.99	ZNF417	0.98	SRC	0.99	SNW1	0.99	TCF12	0.99	ZBTB7A	0.99
ETS1	0.99	NEDD8	0.98	EP300	0.99	CBL	0.99	H2AX	0.99	SGTB	0.99
GATA2	0.99	MTA1	0.98	COPS5	0.99	HNRNPA1	0.99	RAD21	0.99	FOXA2	0.99
APP	0.99	ZBTB16	0.98	GATA1	0.99	ILF3	0.99	GATA1	0.99	SREBF1	0.99
MYC	0.99	CDK9	0.98	H2AX	0.99	PDE4DIP	0.99	USF2	0.99	BATF	0.99
EP300	0.99	PRPF31	0.98	HSPA8	0.99	HNRNPK	0.99	HTT	0.99	EFNA5	0.99
RAD21	0.99	HNRNPM	0.98	PDHA1	0.99	EED	0.99	PCNA	0.99	KRTAP10-3	0.99
AR	0.99	DNM1L	0.98	CASP8	0.99	APC	0.99	UBL4A	0.99	SP2	0.99
UBC	0.99	SNRNP70	0.98	HNF4A	0.99	NFKBIA	0.99	SNCA	0.99	APPBP2	0.99

Supplementary Table 8. The results of removing environmental factors. Model performance and top ten predicted genes after excluding environmental exposure (air pollution) factors in heart failure.

Rank	Original Cohort	Removed air pollution
1	EGR1	UBC
2	CTCF	CTCF
3	ETS1	APP
4	GATA2	GATA2
5	APP	RAD21
6	MYC	EGR1
7	EP300	ETS1
8	RAD21	MYC
9	AR	EP300
10	UBC	AR

Supplementary Table 9. Identified candidate medications and their target for repurposing in heart failure.

Rank	Medication	ATC code	Target genes	Predicted score	Supporting evidence	Known indications
1	Glutamic acid	A09AB01	NAGS, GRIN3A, CPQ, EARS2, SLC1A2, GRIN2C, SLC1A7, GRM4, FOLH1, DNPEP, GRIN2D, GLUD2, AASS, GRIK2, GRIK4, AADAT, GRM7, ENPEP, SLC1A6, BCAT2, GRIK1, GOT2, SLC7A11, GRM8, SLC1A3, GCLC, GPT, GMPS, GPT2, GOT1, GRIA4, NADSYN1, GRIA3, PSAT1, FPGS, GRIN2A, ABAT, GRIK3, BCAT1, GRIA2, GRIA1, GRIN1, GRIK5, TAT, GRM1, EPRS1, ALDH18A1, GLS, GAD2, SLC1A1, GGCX, GLS2, GAD1, ASNS, FTCD, GCLM, OPLAH, GRIN2B, GLUL, GLUD1, PFAS	0.98	response to oxidative stress, regulation of neuron apoptotic process	helps speed the healing of ulcers and helps control schizophrenia
2	Methotrexate	L04AX03	ATIC, TYMS, DHFR	0.97	regulation of response to oxidative stress, regulation of cellular response to oxidative stress	treats cancer, autoimmune diseases and rheumatoid arthritis
3	Topiramate	N03AX11	SCN7A, SCN10A, SCN9A, SCN11A, SCN8A, SCN1A, SCN3A, CACNA1F, SCN4A, CACNB1, GRIK2, GRIK4, CACNA1D, CA3, GRIK1, CA4, SCN2A, CA1, CACNB2, CACNB3, CACNA1E, CACNA1C, CA2, GRIK3, CACNB4, CACNA1S, SCN5A, GRIK5, GABRA1	0.96	regulation of heart rate by cardiac conduction, regulation of ventricular cardiac muscle cell action potential	antiepileptic
4	Probenecid	M04AB01	TAS2R16, SLC22A11, SLC22A8, PANX1, SLC22A6	0.96	positive regulation of macrophage cytokine production	treats chronic gout or gouty arthritis
5	Estradiol acetate	G03CA03	HSD17B2, MT-ATP6, GPER1, NR1I2, BECN1, NCOA2, CHRNA4, ESR2, ESR1, ESRRG	0.96	positive regulation of cardiac vascular smooth muscle cell differentiation, positive regulation of apoptotic signaling pathway	reduces symptoms of menopause
6	Caffeine	N06BC01	PDE7B, PDE8B, PDE11A, PDE6B, PDE1C, PDE1A, PDE5A, PDE4B, ADORA3, PDE6A, PDE10A, ADORA2B, PDE6C, PDE4A, ADORA2A, PDE4C, PDE1B, PDE2A, PIK3CD, PDE8A, ADORA1, PIK3CB, PDE9A, PRKDC, ITPR3, ITPR2, PDE3B, PIK3CA, PDE7A, PDE3A, RYR1, PDE4D, ITPR1, ATM	0.95	regulation of cardiac muscle cell contraction, heart contraction	pain relief product
7	Cannabidiol	N03AX24	TRPV2, TRPV3, TRPV4, TRPA1, GPR55, PTGS1, CACNA1G, HTR1A, GLRA3, CACNA1H, OPRD1, CYP1B1, TRPV1, CYP1A2, CACNA1I, NQO1, CYP3A5, GPR18, NAAA, CYP2D6, CYP17A1, CYP3A7, GPX1, GPR12, CNR2, CAT, IDO1, ACAT1, ADORA1, TRPM8, CNR1, CHRNA7, HMGCR, GLRA1, GSR, OPRM1, HTR2A, VDAC1, PPARG, PTGS2, SOD1, GLRB, HTR3A	0.95	regulation of heart contraction, cellular response to oxidative stress	treats certain types of epilepsy such as Lennox-Gastaut syndrome
8	Prasterone	A14AA07	GABRP, GABRG3, GRIN3A, GRIN3B, GABRQ, GRIN2C, SIGMAR1, GABRA5, GABRA2, GABRB1, GRIN2D, GABRA4, GABRG1, GRIN2A, GABRG2, GABRD, GABRB2, GABRE, GRIN1, GABRA3, NR1I2, NR1I3, PPARA, GABRB3, AR, GABRA6, GRIN2B, ESR2, GABRA1, ESR1	0.95	regulation of epithelial cell apoptotic process	improves symptoms of lupus and erection in men
9	Glycine	B05CX03	GLYATL2, GLYATL1, AGXT2, GRIN3B, GRIN2C, GLRA3, GLYAT, GATM, GPR18, GRIN2A, PIPOX, GLRA2, ALAS2, SHMT1, GCAT, GNMT, GARS1, GLRA1, ALAS1, GSS, BAAT, SHMT2, GLRB, AGXT	0.95	mitochondrial gene expression	antioxidant and anti-inflammatory
10	Ranolazine	C01EB18	KCNJ14, SCN7A, SCN10A, SCN9A, SCN11A, SCN8A, SCN1A, SCN3A, SCN4B, SCN1B, CACNA1F, SCN4A, KCNJ12, CACNB1, KCNJ4, CACNA1D, ADRA1D, SCN2A, CACNB2, CACNB3, CACNA1C, ADRA1B, ADRA1A, CACNB4, CACNA1S, SCN5A, SCN2B, KCNJ2, SCN3B, ADRB1	0.95	regulation of ventricular cardiac muscle cell action potential, positive regulation of heart rate by epinephrine-norepinephrine	treats chest pain related to heart

Supplementary Table 10. The results of validating methotrexate in heart failure using survival models. n is the number of patients taking the medication. The baseline is heart failure with rheumatoid arthritis.

Medication	n	Sex (female%)	Ethnicity (white%)	Age Median[Q1;Q3]	BMI Median[Q1;Q3]
Methotrexate	121	73 (60.33%)	118 (97.5%)	61[56;66]	28.6[24.1;32.3]
Sulfasalazine	27	14 (51.8%)	27 (100%)	61[58;66]	26.9[23.0;32.2]
Hydroxychloroquine	33	27 (81.8%)	32 (96.9%)	62[54;68]	30.6[26.3;36.6]

Supplementary Table 11. The survival probability and hazard ratio for predicted medications for heart failure (HF). The calculated survival probability at 5 years and at 10 years are shown. The extracted cohort includes individuals with HF and rheumatoid arthritis.

Group	HR [95% CI]	Survival probability at 5 years [95% CI]	Survival probability at 10 years [95% CI]	p value
Methotrexate (n=121)	0.65 [0.48, 0.89]	0.92 [0.90, 0.95]	0.84 [0.79, 0.89]	0.01
Hydroxychloroquine (n=33)	1.43 [0.88, 2.32]	0.82 [0.78, 0.91]	0.67 [0.58, 0.81]	0.14
Sulfasalazine (n=27)	1.64 [1.0, 2.69]	0.80 [0.77, 0.84]	0.62 [0.57, 0.69]	0.05

Supplementary Table 12. CoxPH model regression on BioVU medication data for subjects with both Rheumatoid Arthritis (RA) and Heart Failure (HF) diagnoses. Endpoint was defined as 'adverse event' observing either all-cause death or NYHA class 3 or 4. HF diagnosis was defined via ICD10 in range I50 and ICD9 428. RA was defined as ICD10 M06 and ICD9 714. Cox model was mapping survival time of adverse event to medication status, controlling for Age, Sex and body mass index (BMI).

	term	estimate	std.error	statistic	p.value	conf.low	conf.high	significant
1	MedicationMethotrexate	0.71	0.18	-1.98	0.05	0.50	1.00	*
2	MedicationHydroxychloroquine	1.09	0.17	0.49	0.62	0.78	1.51	
3	MedicationSulfasalazine	1.05	0.51	0.09	0.93	0.39	2.82	
4	SEXM	1.33	0.11	2.48	0.01	1.06	1.66	*
5	AGE	1.01	0.00	2.94	0.00	1.00	1.02	**
6	bmi	1.01	0.01	2.00	0.05	1.00	1.02	*

Supplementary Table 13. Identified candidate medications and their target for repurposing in atrial fibrillation.

Rank	Medication	ATC code	Target genes	Predicted score	Supporting evidence	Known indications
1	Methotrexate	L04AX03	ATIC, TYMS, DHFR	0.80	regulation of response to oxidativestress, regulation of cellular response to oxidative stress	Treats cancer, autoimmune diseases and rheumatoid arthritis
2	Zonisamide	N03AX15	CA7, CA5B, CA13, SCN9A, SCN11A, CACNA1G, SCN1A, SCN3A, SCN4B, SCN1B, CACNA1H, SCN4A, CA11, CACNA1I, CA3, CA5A, CA12, CA4, SCN2A, CA1, CA14, CA6, CA10, CA2, CA9, MAOA, MAOB, SCN5A, SCN2B, CA8, SCN3B	0.80	regulation of ventricular cardiac muscle cell membrane repolarization, regulation of ventricular cardiac muscle cell membrane depolarization	antiepileptic
3	Probenecid	M04AB01	TAS2R16, SLC22A11, SLC22A8, PANX1, SLC22A6	0.79	positive regulation of macrophage cytokine production	treats chronic gout or gouty arthritis
4	Estradiol valerate	G03CA03	HSD17B2, MT-ATP6, GPER1, NR1I2, BECN1, NCOA2, CHRNA4, ESR2, ESR1, ESRRG	0.79	positive regulation of cardiac vascular smooth muscle cell differentiation, positive regulation of apoptotic signaling pathway	treating conditions related to low estrogen levels, such as menopausal symptoms and certain cancers
5	Estradiol cypionate	G03CA03	HSD17B2, MT-ATP6, GPER1, NR1I2, BECN1, NCOA2, CHRNA4, ESR2, ESR1, ESRRG	0.79	positive regulation of cardiac vascular smooth muscle cell differentiation, positive regulation of apoptotic signaling pathway	treatment of hypoestrogenism
6	Estradiol acetate	G03CA03	HSD17B2, MT-ATP6, GPER1, NR1I2, BECN1, NCOA2, CHRNA4, ESR2, ESR1, ESRRG	0.79	cardiac vascular smooth muscle cell differentiation, positive regulation of apoptotic signaling pathway	reduces symptoms of menopause
7	Glycine	B05CX03	GLYATL2, GLYATL1, AGXT2, GRIN3B, GRIN2C,GLRA3, GLYAT, GATM, GPR18, GRIN2A, PIPOX, GLRA2, ALAS2, SHMT1, GCAT, GNMT, GARS1,GLRA1, ALAS1, GSS, BAAT, SHMT2, GLRB, AGXT	0.77	mitochondrial gene expression	used as irrigation during surgery
8	Glutamic acid	A09AB01	NAGS, GRIN3A, CPQ, EARS2, SLC1A2, GRIN2C,SLC1A7, GRM4, FOLH1, DNPEP, GRIN2D, GLUD2,AASS, GRIK2, GRIK4, AADAT, GRM7, ENPEP,SLC1A6, BCAT2, GRIK1, GOT2, SLC7A11, GRM8, SLC1A3, GCLC, GPT, GMPS, GPT2, GOT1, GRIA4, NADSYN1, GRIA3, PSAT1, FPGS, GRIN2A, ABAT, GRIK3, BCAT1, GRIA2, GRIA1, GRIN1, GRIK5, TAT,GRM1, EPRS1, ALDH18A1, GLS, GAD2, SLC1A1, GGCX, GLS2, GAD1, ASNS, FTCD, GCLM, OPLAH,GRIN2B, GLUL, GLUD1, PFAS	0.76	response to oxidative stress, regulation of apoptotic process	helps speed the healing of ulcers and helps control schizophrenia
9	Acamprosate	N07BB04	GABRP, GABRG3, GRIN3A, GRIN3B, GABRQ, GRIN2C, GABRA5, GABRA2, GABRB1, GRIN2D, GABRA4, GABRG1, GRIN2A, GABRG2, GABRD, GRM5, GABRB2, GABRE, GRIN1, GABRA3	0.76	abnormal nervous system electrophysiology, positive regulation of cell communication	reduces alcoholism cravings
10	Pentobarbital	N05CA01	GABRP, GABRG3, GRIN3A, GRIN3B, GABRQ, GRIN2C, GABRA5, GABRA2, GABRB1, GRIN2D, GRIK2, GABRA4, GABRG1, GRIN2A, GABRG2, GABRD, GRIA2, GABRB2, GABRE, GRIN1, GABRA3, CHRNA7, NR1I2, GABRB3, CHRNA4, GABRA6, GRIN2B, GABRA1	0.75	positive regulation of cell communication, abnormal nervous system electrophysiology	treate seizures

Supplementary Table 14. Outcomes for methotrexate and other drugs in atrial fibrillation. n is the number of patients taking the medication. The baseline is atrial fibrillation with rheumatoid arthritis.

Medication	n	Sex (female%)	Ethnicity (white%)	Age Median[Q1;Q3]	BMI Median[Q1;Q3]
Methotrexate	170	79 (57.1%)	167 (98.2%)	71.0[64.0;75.6]	28.0[25.4;31.9]
Sulfasalazine	46	23 (50.0%)	45 (97.8%)	71.0[67.0;76.0]	28.7[24.6;31.9]
Hydroxychloroquine	39	29 (74.4%)	37 (94.9%)	69.0[66.5;75.0]	30.9[26.1;39.2]

Supplementary Table 15. Survival probability and hazard ratios for medications in atrial fibrillation (AF). The calculated survival probability at 5 years and at 10 years are shown. The extracted cohort includes individuals with AF and rheumatoid arthritis.

Group	HR [95% CI]	Survival probability at 5 years [95% CI]	Survival probability at 10 years [95% CI]	p value
Methotrexate (Baseline, n= 170)	1.0	0.85 [0.79, 0.94]	0.70 [0.58, 0.86]	NA
Hydroxychloroquine (n=39)	1.76 [0.89, 3.49]	0.75 [0.65, 0.86]	0.54 [0.35, 0.69]	0.10
Sulfasalazine (n=46)	1.01 [0.48, 2.10]	0.84 [0.78, 0.92]	0.68 [0.55, 0.82]	0.90

Supplementary Table 16. Identified candidate medications and their target for re-purposing in myocardial infarction.

Rank	Medication	ATC code	Target genes	Predicted score	Supporting evidence	Known indications
1	Vorinostat	L01XH01	HDAC8, HDAC2, HDAC6,HDAC1, HDAC3	0.99	positive regulation of response to oxidative stress, negative regulation of programmed cell death	treats cutaneous T-cell lymphoma (CTCL)
2	Podofilox	D06BB04	TUBB,TUBA4A, TOP2A	0.99	cellular component disassembly involved in execution phase of apoptosis	treats molluscum contagiosum
3	Mecasermin rinfabate	H01AC05	IGF2R, IGF1R, INSR	0.99	regulation of programmed cell death, heart development	treats growth failure in children with severe primary IGF-1 deficiency
4	Ingenol mebutate	D06BX02	PRKCD,PRKCA	0.99	intrinsic apoptotic signaling pathway in response to oxidative stress, negative regulation of inflammatory response	treats actinic keratosis
5	Dimethyl fumarate	L04AX07	RELA,KEAP1	0.99	negative regulation of extrinsic apoptotic signaling pathway, cellular response to oxidative stress	treats the relapsing forms of multiple sclerosis
6	Glucosamine	M01AX05	MMP9,IFNG NFKB2,TNF	0.99	extrinsic apoptotic signaling pathway via death domain receptors, apoptotic mitochondrial changes	might provide some pain relief for people with osteoarthritis
7	Lasofoxifene	G03XC03	CNR2,ESR2, ESR1	0.99	inflammatory response, negative regulation of muscle cell apoptotic process	prevention and treatment of osteoporosis
8	Dexrazoxane	V03AF02	TOP2B, TOP2A	0.99	execution phase of apoptosis, cellular component disassembly involved in execution phase of apoptosis	decreases damage to the skin and tissues that may be caused when an anthracycline chemotherapy medication
9	Omega-3	C10AX06	PPARA,PPARG, SREBF1	0.99	regulation of apoptotic process, adaptation cardiac muscle tissue growth, regulation of cardiac muscle tissue growth, cardiac muscle cell development	decreases the amount of triglycerides and other fats made in the liver
10	Auranofin	M01CB03	PRDX5, IKBKB	0.99	inflammatory response	treats rheumatoid arthritis

Supplementary Table 17. The demographics and the imaging characteristics of the validation cohort from the UK Biobank. All the individuals (n=2,451) have cardiac magnetic resonance imaging (CMR). AF: atrial fibrillation, MI: myocardial infarction, HF: heart failure, HCM: hypertrophic cardiomyopathy, DCM: dilated cardiomyopathy, BMI: body mass index, BSA: body surface area.

**Baseline characteristics**	**AF** (n=1,173)	**MI** (n=756)	**HF** (n=274)	**HCM** (n=25)	**DCM** (n=34)
Sex Males(%)	806 (68.71%)	613 (81.08%)	189 (68.79%)	17 (68.00%)	23 (67.64%)
Ethnicity White(%)	1151 (98.12%)	726 (96.03%)	254 (96.71%)	25 (100%)	34 (100%)
Age at time of MRI (years) Median[Q1;Q3]	75.0 [70.0; 78.0]	74.0 [69.0; 78.0]	75.0 [70.0; 78.0]	69.0 [62.0; 77.0]	74.0 [71.0; 77.7]
Body Mass Index (kg/m^2) Median[Q1;Q3]	27.10 [24.50; 30.30]	27.50 [24.77; 30.02]	27.60 [24.62; 30.75]	27.30 [24.50; 31.20]	28.20 [25.00; 30.30]
Body Surface Area (m^2) Median[Q1;Q3]	1.97 [1.83; 2.13]	1.95 [1.82; 2.10]	1.97 [1.82; 2.14]	1.91 [1.80; 2.02]	1.96 [1.82; 2.16]
**CMR**	Median[Q1;Q3]	Median[Q1;Q3]	Median[Q1;Q3]	Median[Q1;Q3]	Median[Q1;Q3]
Left ventricle end systolic volume (ml)	33.41[27.20;41.03]	34.69[27.82;44.11]	37.08[30.15;47.01]	31.23[24.61;39.68]	42.57[36.83;53.18]
Left ventricle end diastolic volume (ml)	77.7[66.44;87.77]	81.15[71.17;93.17]	79.19[68.36;92.87]	76.55[65.36;89.73]	83.38[70.34;98.24]
Left ventricle systolic volume (ml)	42.94[35.57;50.49]	45.14[39.79;51.09]	41.91[35.15;48.36]	45.94[38.9;50.7]	40.63[32.86;48.99]
Left ventricle cardiac output (ml)	5.22[4.41;6.15]	5.07[4.4;5.84]	5.0[4.27;5.96]	5.47[4.31;5.91]	5.03[4.45;5.95]
Left ventricle ejection fraction (%)	56.88[49.42;62.09]	56.63[50.18;61.97]	52.89[46.83;58.86]	59.96[53.09;63.1]	51.84[42.48;55.52]
Left ventricle mass (g)	46.30[40.48;52.26]	48.53[43.74;53.88]	47.70[41.77;54.69]	58.87[47.49;67.81]	44.41[39.38;57.34]
Right ventricle end systolic volume (ml)	39.05[32.16;46.78]	35.25[30.39;42.47]	37.55[30.52;46.7]	33.48[26.5;39.08]	41.33[32.15;52.28]
Right ventricle end diastolic volume (ml)	81.78[72.3;93.85]	81.19[72.01;90.93]	80.03[68.97;91.83]	70.22[66.92;82.21]	78.61[67.75;91.4]
Right ventricle systolic volume (ml)	42.98[35.12;51.12]	45.36[39.27;51.60]	42.31[34.78;49.47]	43.47[34.01;48.05]	39.34[31.64;47.33]
Right ventricle ejection fraction (%)	56.38[51.76;60.02]	53.68[47.54;58.52]	53.96[48.34;59.28]	56.54[52.93;60.52]	50.11[39.91;55.91]
Left atrium maximum volume (ml)	50.25[38.7;66.78]	43.06[33.62;52.68]	45.48[36.9;61.13]	56.98[40.41;62.08]	56.60[39.73;70.81]
Left atrium minimum volume (ml)	25.51[16.56;48.3]	19.26[13.63;25.46]	22.18[16.09;38.33]	27.53[18.33;36.56]	28.50[14.86;53.6]
Right atrium maximum volume (ml)	54.75[42.54;70.08]	44.24[36.41;55.39]	49.44[37.94;62.99]	43.31[36.79;52.98]	53.62[37.2;73.97]
Right atrium minimum volume (ml)	32.8[22.9;48.30]	24.53[18.88;31.82]	28.22[21.05;41.28]	23.01[18.71;29.98]	28.55[19.11;48.90]
Descending aorta distensibility (10^{-3} .mmHg^{-1})	1.49[1.07;2.13]	1.66[1.15;2.3]	1.51[1.06;2.12]	2.07[1.28;2.53]	1.95[1.10;2.43]
Ascending aorta distensibility (10^{-3} .mmHg^{-1})	1.17[0.83;1.7]	1.19[0.84;1.72]	1.13[0.82;1.74]	1.51[1.07;2.33]	1.30[0.74;2.52]
Global longitudinal strain (%)	-16.59[-19.18;-13.37]	-17.45[-19.48;-15.15]	-16.21[-18.2;-13.42]	-16.48[-17.76;-13.25]	-15.07[-17.44;-11.38]
Circumferential peak diastolic strain rate (s^{-1})	1.84[1.31;2.43]	1.85[1.28;2.44]	1.60[1.14;2.12]	2.09[1.43;2.60]	1.38[0.88;1.81]
Longitudinal peak diastolic strain rate (s^{-1})	1.41[1.08;1.85]	1.45[1.11;1.86]	1.30[0.96;1.77]	1.28[1.03;1.89]	0.91[0.75;1.16]
Radial peak diastolic strain rate (s^{-1})	-4.86[-6.43;-3.37]	-4.91[-6.5;-3.45]	-4.14[-6.07;-3.02]	-5.73[-7.06;-4.29]	-3.33[-4.44;-2.79]
Maximum wall thickness (mm)	10.37[9.32;11.56]	10.28[9.31;11.63]	10.48[9.23;11.76]	14.17[10.94;15.91]	9.86[8.53;11.38]

Supplementary Table 18. The predicted medications for heart failure. The top ten predicted medications for heart failure in in the original cohort and in the independent UK Biobank cohort, with their ranks.

Rank	Original Cohort	Independent validation
1	Glutamic acid	Methotrexate
2	Methotrexate	Topiramate
3	Topiramate	Prednisolone phosphate
4	Probenecid	Budesonide
5	Estradiol acetate	Prednisone
6	Caffeine	Estradiol acetate
7	Cannabidiol	Estradiol dienanthate
8	Prasterone	Estradiol valerate
9	Glycine	Estradiol cypionate
10	Ranolazine	Estradiol benzoate

Supplementary Table 19. The predicted genes for heart failure. Top 10 predicted genes in the original cohort and in the independent UK Biobank cohort, with their ranks.

Rank	Original Cohort	Independent validation
1	EGR1	GATA2
2	CTCF	CTCF
3	ETS1	UBC
4	GATA2	ETS1
5	APP	EGR1
6	MYC	APP
7	EP300	MYC
8	RAD21	EP300
9	AR	AR
10	UBC	RAD21

Supplementary Table 20. Impact of comorbidities on predicted genes. This table compares the top predicted genes with and without patients who had common comorbidities (hypertension n = 20, diabetes n = 7, chronic kidney disease (CKD n = 11), chronic obstructive pulmonary disease (COPD n = 15)) . The same set of genes was identified in both analyses, with only minor differences in their ranking order.

Rank	Original Cohort	Removed Comorbidities
1	EGR1	CTCF
2	CTCF	UBC
3	ETS1	GATA2
4	GATA2	ETS1
5	APP	EGR1
6	MYC	APP
7	EP300	MYC
8	RAD21	RAD21
9	AR	EP300
10	UBC	AR

Supplementary Table 21. Medications used to train the machine learning model for predicting potential associations between medications and heart failure.

Medication indicated for the treatment of HF	Medication contraindicated for the treatment of HF
"Adomiparin", "Parnaparin", "Evolocumab", "Valsartan", "Bivalirudin", "Prasugrel", "Esmolol", "Fondaparinux", "Reteplase", "Nitroglycerin", "Phenprocoumon", "Alteplase", "Ardeparin", "Irbesartan", "Eptifibatide", "Tirofiban", "Dalteparin", "Dicoumarol", "Abciximab","Tenecteplase", "Heparin", "Alirocumab", "Atenolol", "Morphine", "Ticagrelor", "Vorapaxar", "Candesartan", "Captopril", "Lisinopril", "Quinapril", "Acetylsalicylic acid", "Enalapril", "Metoprolol", "Clopidogrel", "Losartan", "Heparin"	"Metamfetamine", "Testosterone cypionate", "Testosterone enanthate", "Sildenafil", "Saxagliptin", "Levodopa", "Furosemide", "Phenobarbital", "Guanfacine", "Flecainide", "Indomethacin", "Lovastatin", "Progesterone", "Testosterone", "Simvastatin", "Dipyridamole", "Pioglitazone", "Nefazodone", "Paclitaxel", "Phenytoin", "Liothyronine", "Levothyroxine", "Diclofenac", "Estradiol", "Meclofenamic acid", "Estradiol cypionate", "Estradiol valerate", "Digoxin", "Ribavirin", "Inositol nicotinate", "Levomefolic acid", "Acyclovir", "Sorafenib", "Metformin", "Sodium citrate", "Inositol"

Supplementary Table 22. Medications used to train the machine learning model for predicting potential associations between medications and atrila fibrillation.

Medication indicated for the treatment of AF	Medication contraindicated for the treatment of AF
"Dabigatran", "Sotalol", "Warfarin", "Heparin", "Atenolol", "Diltiazem", "Timolol", "Bisoprolol", "Carvedilol", "Propafenone", "Edoxaban", "Betrixaban", "Rivaroxaban", "Apixaban", "Labetalol", "Dofetilide", "Metoprolol", "Propranolol", "Amiodarone", "Clopidogrel", "Quinidine", "Flecainide", "Dronedarone", "Digoxin", "Verapamil", "Procainamide", "Nadolol","Dipyridamole","Esmolol","Acetyldigitoxin"	"Bupivacaine", "Methylphenidate", "Cholecalciferol", "Lacosamide", "Alendronic acid", "Dobutamine","Ergocalciferol", "Lidocaine", " Fidaxomicin", "Azithromycin", "Fusidic acid", "Amoxicillin", "Naproxen", "Ibuprofen", "Oxytetracycline", "Tetracycline", "Disopyramide", "Quinine", "Epinephrine", "Conivaptan","Cetirizine", "Dorzolamide","Pantoprazole" ,"Loratadine","Raltitrexed","Methadone", "Erythromycin", "Clarithromycin"

Supplementary Table 23. Medications used to train the machine learning model for predicting potential associations between medications and myocardial infarction.

Medication indicated for the treatment of MI	Medication contraindicated for the treatment of MI
"Adomiparin", "Heparin", "Parnaparin", "Evolocumab", "Bivalirudin", "Prasugrel", "Esmolol", "Fondaparinux", "Reteplase","Nitroglycerin", "Phenprocoumon", "Alteplase", "Ardeparin", "Irbesartan", "Eptifibatide", "Tirofiban", "Dalteparin", "Dicoumarol", "Abciximab","Tenecteplase", "Heparin", "Alirocumab", "Atenolol", "Morphine", "Ticagrelor", "Vorapaxar", "Candesartan", "Captopril", "Lisinopril", "Quinapril", "Acetylsalicylic acid", "Enalapril", "Metoprolol", "Clopidogrel", "Valsartan", "Losartan"	"Metamfetamine", "Testosterone cypionate", "Testosterone enanthate", "Sildenafil", "Saxagliptin", "Levodopa", "Furosemide", "Phenobarbital", "Guanfacine", "Flecainide", "Indomethacin", "Lovastatin", "Progesterone", "Testosterone", "Simvastatin", "Dipyridamole", "Pioglitazone", "Nefazodone", "Paclitaxel", "Phenytoin", "Liothyronine", "Levothyroxine", "Diclofenac", "Estradiol", "Meclofenamic acid", "Estradiol cypionate", "Estradiol valerate", "Digoxin", "Ribavirin", "Inositol nicotinate", "Levomefolic acid", "Acyclovir", "Sorafenib", "Metformin", "Sodium citrate", "Inositol"
