# Supplementary material for: A multimodal vision knowledge graph of cardiovascular disease
Source: Nat Cardiovasc Res. 2025 Dec 29;5(1):18–33. doi: 10.1038/s44161-025-00757-4 (PMC12811117; doi:10.1038/s44161-025-00757-4)
Supplement: Supplementary file 2 — Reporting Summary [file 44161_2025_757_MOESM2_ESM.pdf]

nature portfolio

Corresponding author(s): Declan P O'Regan

Last updated by author(s): Oct 30, 2025

Reporting Summary

Nature Portfolio wishes to improve the reproducibility of the work that we publish. This form provides structure for consistency and transparency in reporting. For further information on Nature Portfolio policies, see our Editorial Policies and the Editorial Policy Checklist.

Statistics

For all statistical analyses, confirm that the following items are present in the figure legend, table legend, main text, or Methods section.

n/a	Confirmed	
☐	☒	The exact sample size (*n*) for each experimental group/condition, given as a discrete number and unit of measurement
☒	☐	A statement on whether measurements were taken from distinct samples or whether the same sample was measured repeatedly
☐	☒	The statistical test(s) used AND whether they are one- or two-sided<br>*Only common tests should be described solely by name; describe more complex techniques in the Methods section.*
☐	☒	A description of all covariates tested
☐	☒	A description of any assumptions or corrections, such as tests of normality and adjustment for multiple comparisons
☐	☒	A full description of the statistical parameters including central tendency (e.g. means) or other basic estimates (e.g. regression coefficient) AND variation (e.g. standard deviation) or associated estimates of uncertainty (e.g. confidence intervals)
☐	☒	For null hypothesis testing, the test statistic (e.g. *F*, *t*, *r*) with confidence intervals, effect sizes, degrees of freedom and *P* value noted<br>*Give P values as exact values whenever suitable.*
☒	☐	For Bayesian analysis, information on the choice of priors and Markov chain Monte Carlo settings
☒	☐	For hierarchical and complex designs, identification of the appropriate level for tests and full reporting of outcomes
☒	☐	Estimates of effect sizes (e.g. Cohen's *d*, Pearson's *r*), indicating how they were calculated

Our web collection on statistics for biologists contains articles on many of the points above.

Software and code

Policy information about availability of computer code

Data collection	Data were obtained from the UK Biobank under approved application number 40616. Data access and extraction were performed using the UK Biobank Research Analysis Platform (UK RAP). The study received ethical approval from the National Research Ethics Service (11/NW/0382), and all participants gave written informed consent. Custom Python scripts (Python version 3.10) were used within the RAP environment to filter participants based on imaging availability and clinical diagnoses (ICD-9/ICD-10 codes). Standard libraries such as pandas (v1.5.3) and numpy (v1.24.2) were used for data manipulation. No commercial software was used for data collection.
Data analysis	Custom Python scripts (Python version 3.12.1) and neo4j Desktop (version 5.20.0) were used to run the analysis.

For manuscripts utilizing custom algorithms or software that are central to the research but not yet described in published literature, software must be made available to editors and reviewers. We strongly encourage code deposition in a community repository (e.g. GitHub). See the Nature Portfolio guidelines for submitting code & software for further information.

Data

Policy information about availability of data

All manuscripts must include a data availability statement. This statement should provide the following information, where applicable:

- Accession codes, unique identifiers, or web links for publicly available datasets
- A description of any restrictions on data availability
- For clinical datasets or third party data, please ensure that the statement adheres to our policy

The scripts for data analysis are publicly available at https://github.com/ImperialCollegeLondon/cardioKG(DOI:10.5281/zenodo.16025952). Data from UK Biobank are available for approved research upon application https://www.ukbiobank.ac.uk/enable-your-research/apply-for-access under the terms of the UK Biobank's data access policy.

Research involving human participants, their data, or biological material

Policy information about studies with human participants or human data. See also policy information about sex, gender (identity/presentation), and sexual orientation and race, ethnicity and racism.

Reporting on sex and gender	Sex (biological) was self-reported by participants at recruitment and recorded in the UKBiobank dataset. In our study, sex (males n = 4893 and females n = 4691) was included as a covariate and incorporated into the model as part of the node feature set for each individual. It was also adjusted for in downstream analyses, such as survival analysis for drug repurposing outcomes. Gender identity was not available in the UKBiobank dataset, and therefore was not analyzed in this study.
Reporting on race, ethnicity, or other socially relevant groupings	Ethnicity was self-reported by participants in the UK Biobank at baseline. In our study cohort, over 90% of participants were of White European ancestry, reflecting the broader UK Biobank population. Due to this limited ethnic diversity, subgroup analyses by ethnicity were not performed. Other socially relevant variables such as socioeconomic status or gender identity were not included in the current analysis.<br><br>To control for potential confounding, we incorporated available demographic and clinical covariates into our graph-based model where applicable. Specifically: Age and sex were included as node-level features in the knowledge graph. Disease and medication nodes were connected via structured diagnostic and treatment codes (ICD, ATC), minimizing subjective or biased inputs. For downstream validation (e.g., survival analysis in the drug repurposing experiment), we adjusted for age and sex to account for their known associations with cardiovascular risk and treatment response.
Population characteristics	The study population was derived from the UK Biobank, a population-based cohort of approximately 500,000 individuals aged 40–69 years at recruitment (2006–2010) across the United Kingdom. For this study, we selected 4,280 participants with both cardiac magnetic resonance (CMR) imaging data and a diagnosis of one of five cardiovascular diseases: heart failure (HF), atrial fibrillation (AF), myocardial infarction (MI), hypertrophic cardiomyopathy (HCM), or dilated cardiomyopathy (DCM), identified via ICD-9/10 codes. An additional 5,304 participants without a cardiovascular diagnosis were included as healthy controls to ensure representation across the phenotypic spectrum.<br><br>The demographic and clinical characteristics of the study population — including age, sex and ethnicity— are provided in Supplementary Table 2. Consistent with the overall UK Biobank cohort, over 90% of participants were of White European ancestry
Recruitment	Participants were not directly recruited for this study. All data were obtained from the UK Biobank, a large population-based cohort of approximately 500,000 individuals aged 40–69 years who were recruited across the United Kingdom between 2006 and 2010. Recruitment was conducted by UK Biobank using population-wide mail invitations, and participation was voluntary following informed consent.
Ethics oversight	The study received ethical approval from the National Research Ethics Service (11/NW/0382)

Note that full information on the approval of the study protocol must also be provided in the manuscript.

Field-specific reporting

Please select the one below that is the best fit for your research. If you are not sure, read the appropriate sections before making your selection.

☒ Life sciences ☐ Behavioural & social sciences ☐ Ecological, evolutionary & environmental sciences

For a reference copy of the document with all sections, see nature.com/documents/nr-reporting-summary-flat.pdf

Life sciences study design

All studies must disclose on these points even when the disclosure is negative.

Sample size	The sample was drawn from the UK Biobank, a population-based cohort of ~500,000 individuals. A sub-cohort who underwent cardiac magnetic resonance (CMR) imaging was used for this study. From this group, we selected 4,280 individuals with imaging data and a diagnosis of one of five target cardiovascular diseases (AF, HF, MI, HCM, or DCM), and an additional 5,304 healthy controls to ensure broad phenotypic

variability. This sample size was determined based on the availability of both imaging and diagnostic information relevant to the target phenotypes.

Over 200,000 image-derived phenotypes (IDPs) were extracted using computer vision pipelines and incorporated into the model. We focused analyses on the three most prevalent conditions (AF, HF, MI) to ensure adequate statistical power and clinical relevance. The sample size is considered sufficient given the large number of quantitative imaging traits per participant and the inclusion of both diseased and healthy individuals for contrastive learning.

Data exclusions

Participants were included if they had (i) available cardiac magnetic resonance (CMR) imaging data and (ii) a documented diagnosis of one of five target cardiovascular diseases: heart failure (HF), atrial fibrillation (AF), myocardial infarction (MI), hypertrophic cardiomyopathy (HCM), or dilated cardiomyopathy (DCM), based on ICD-9/10 codes.We excluded individuals who lacked CMR imaging data or who did not have one of the five specified cardiovascular diagnoses.Rationale:The goal of this study was to investigate the role of imaging-derived phenotypes in predicting gene–disease associations. Including only participants with both imaging and disease diagnosis ensured that the model could leverage structural and functional cardiac traits as features directly relevant to disease status.

Replication

To verify the reproducibility of our drug repurposing experiment, we performed survival analysis on an independent validation cohort of 489 participants who did not have cardiac imaging data. This cohort was used to assess whether drugs prioritized by our predictive model (based on imaging-derived phenotypes and gene–disease associations) were associated with improved survival in a separate population. The consistent patterns observed supported the validity and generalisability of the drug repurposing predictions. Furthermore, We evaluated the reproducibility of our findings in an independent UK Biobank cohort of 33,822 participants with CMR imaging, including 1,173 with AF, 756 with MI, 274 with HF, 25 with HCM, and 34 with DCM. All reproducibility checks were successful.

Randomization

Yes, randomization was applied via a fixed random seeds, ensuring consistent and repeatable results across model runs.

Blinding

Blinding was not applicable in this study. All data were pre-existing and sourced from the UK Biobank, and no new data collection was performed by the investigators. Group allocation (e.g., disease vs. control) was determined based on structured clinical codes (ICD-9/ICD-10) and imaging availability prior to analysis. Since the analysis was entirely computational and automated using predefined criteria and scripts, investigator blinding was not relevant.

Reporting for specific materials, systems and methods

We require information from authors about some types of materials, experimental systems and methods used in many studies. Here, indicate whether each material, system or method listed is relevant to your study. If you are not sure if a list item applies to your research, read the appropriate section before selecting a response.

Materials & experimental systems

n/a	Involved in the study	
☒	☐	Antibodies
☒	☐	Eukaryotic cell lines
☒	☐	Palaeontology and archaeology
☒	☐	Animals and other organisms
☒	☐	Clinical data
☒	☐	Dual use research of concern
☒	☐	Plants

Methods

n/a	Involved in the study	
☒	☐	ChIP-seq
☒	☐	Flow cytometry
☒	☐	MRI-based neuroimaging

Plants

Seed stocks

Report on the source of all seed stocks or other plant material used. If applicable, state the seed stock centre and catalogue number. If plant specimens were collected from the field, describe the collection location, date and sampling procedures.

Novel plant genotypes

Describe the methods by which all novel plant genotypes were produced. This includes those generated by transgenic approaches, gene editing, chemical/radiation-based mutagenesis and hybridization. For transgenic lines, describe the transformation method, the number of independent lines analyzed and the generation upon which experiments were performed. For gene-edited lines, describe the editor used, the endogenous sequence targeted for editing, the targeting guide RNA sequence (if applicable) and how the editor was applied.

Authentication

Describe any authentication procedures for each seed stock used or novel genotype generated. Describe any experiments used to assess the effect of a mutation and, where applicable, how potential secondary effects (e.g. second site T-DNA insertions, mosiacism, off-target gene editing) were examined.
